# Within a Heartbeat: A Machine Learning Approach Using VCG for Heart Failure Assessment in Sinus Rhythm

**DOI:** 10.3390/jpm16090480

**Published:** 2026-09-17

**Authors:** Ioannis Marios Karagiannis, Athanasios Samaras, Dimitrios Filos, George Giannakoulas, Ioanna Chouvarda

**Affiliations:** 1 Lab of Computing, Medical Informatics, and Biomedical-Imaging Technologies, School of Medicine, Aristotle University of Thessaloniki, 541 24 Thessaloniki, Greece; dimfilos@auth.gr; 2 Second Department of Cardiology, Ippokratio General Hospital, Aristotle University of Thessaloniki, 546 42 Thessaloniki, Greece; ath.samaras.as@gmail.com; 3 First Department of Cardiology, AHEPA University Hospital, Aristotle University of Thessaloniki, 546 36 Thessaloniki, Greece; g.giannakoulas@gmail.com

**Keywords:** heart failure (HF), left ventricular ejection fraction (LVEF), vectorcardiogram (VCG), sinus rhythm (SR), machine learning, biomedical signal processing, continuous wavelet transform (CWT), heart-rate variability (HRV), support vector machine (SVM)

## Abstract

**Background/Objectives:** Heart failure (HF) is associated with structural changes such as ventricular dilation, hypertrophy and fibrosis that alter conduction pathways and leave characteristic traces in the electrical activity of each heartbeat. Individuals diagnosed with HF exhibit a range of clinical manifestations and require diverse therapeutic interventions. Nevertheless, an accurate and timely diagnosis must precede any therapeutic approach. Patients with HF and a high ejection fraction (EF) frequently remain undiagnosed, as preliminary medical assessments cannot effectively distinguish them from asymptomatic individuals. **Methods:** In this work, vectorcardiography (VCG) data (277 samples) were used from the PTB and MUSIC databases, with a primary endpoint of identifying individuals with a high likelihood of HFpEF (EF ≥ 50%) compared to healthy subjects to help mitigate overlooked cases and lay the groundwork for patient-centered care. The second endpoint was to screen whether an individual was healthy or had an HF subtype (HFrEF or HFpEF), making it relevant as a potential triage support in clinical practice. To achieve these objectives, heart-rate variability (HRV) features and continuous wavelet transform (CWT) features for each QRS complex and ST-T segment were extracted to train traditional machine learning (ML) models: support vector machine (SVM), random forest (RF), and eXtreme gradient boosting (XGBoost). **Results:** Across 100 random train/test splits, the first endpoint achieved Recall = 0.88 and AUROC = 0.98. For the second endpoint, aggregated across three internal test sets, the classification of healthy individuals, HFrEF and HFpEF resulted in Recall = 0.88, AUROC = 0.99, Recall = 0.76, AUROC = 0.87, Recall = 0.60 and AUROC = 0.79, respectively. **Conclusions:** These findings suggest that ECG/VCG-based machine learning approaches may contribute to HF subtype stratification and have the potential to broaden access to precision diagnosis and personalized medicine. As healthy and HF patients come from different databases and the study lacks external validation, findings should be interpreted with care.

## 1. Introduction

Heart failure (HF) is a clinical syndrome caused by structural and/or functional abnormalities of the heart, leading to impaired ventricular filling and/or blood ejection [1]. It affects approximately 64 million people globally, and its already high worldwide prevalence (over 10% in those over 70 years old) is expected to rise further due to an aging population, improved treatment and improved survival rates [2], underpinning the need for timely diagnosis and risk management. Among the challenges in HF is the heterogeneity of the disease. Clinically relevant patient categories are defined based on the ejection fraction (EF), which is assessed via transthoracic echocardiography and calculated as the ratio of stroke volume to end-diastolic volume [3]. Patients exhibiting preserved EF (HFpEF, EF ≥ 50%) receive different treatments than those with reduced EF (HFrEF, EF ≤ 40%) [4]. However, there is also an EF zone between 41 and 49% called mildly reduced EF (HFmrEF), where patients present characteristics more similar to those of HFrEF and respond better to HFrEF than HFpEF therapies [4]. Studies have also suggested that HFmrEF is a transitional state from HFpEF to HFrEF, and it is referred to as a gray zone [4]. HFpEF comprises approximately 50% of heart failure cases [2]. Despite the preserved ejection fraction, HFpEF is a condition related to high morbidity, mortality, and healthcare costs, making its timely identification clinically essential.

In clinical practice, ECG plays a supportive role in the diagnosis of HFpEF, helping to evaluate patients for referral to B-type natriuretic peptide (BNP)/NT-proBNP tests and further echocardiographic examination. According to guidelines, a normal or not particularly abnormal ECG and a low BNP/NT-proBNP value lower the suspicion of HFpEF and lead to underdiagnosed cases [4]. In order to further investigate ECG value, previous work [5] extensively studied traditional ECG with respect to structural heart abnormalities and low specificity for functional abnormalities, highlighting the need to develop novel methods for the evaluation of HF. Differences in ECG and HF subtypes were examined in [6], suggesting that ventricular repolarization and ventricular depolarization markers can distinguish between patients with HFrEF and HFpEF, showcasing the role of ECG in the personalized risk assessment of HF subtypes. Differences do occur—not only among subtypes but also within the same subtype, resulting in different phenotypes that require precise medical interventions [7,8,9].

Recent findings [10,11] suggest that HFpEF is often underdiagnosed using standard guidelines, indicating a pressing need for other non-invasive diagnostic devices and tools, including AI-DSS tools.

Previous studies have addressed this problem using different AI approaches to improve diagnosis, focusing mainly on the HFpEF vs. healthy classification. In [12], an ML model (XGBoost) was developed by combining demographic, clinical, and echocardiography data to detect HFpEF vs. controls, demonstrating strong performance, with an AUROC of 0.89 (95% CI: 0.85–0.93) in an external validation dataset of 3349 samples. In [13], an AI-ECG model was implemented based on a CNN solution, where patients were classified as HFpEF (HFA-PEFF score ≥ 5) or controls, achieving a good discriminative performance of AUROC = 0.81 (95% CI: 0.80–0.82) in an internal validation set of 2167 samples. Recently, in [14], a ResNet-based deep learning model (ECG-AI) was extensively validated in 100.000 recordings for early identification of HFpEF based on ECG obtained up to six months prior to diagnosis, exhibiting impressive results, with AUROC = 0.79 (95% CI: 0.76–0.82). Similarly, in [15], a CNN-based model demonstrated competitive results in external validation (samples = 203), with an AUROC of 0.80 (95% CI: 0.74–0.86).

Studies that address HFrEF vs. healthy or HF subtype stratification using ECG have received less attention. For HFrEF detection against healthy controls, a CNN model [16] achieved an AUROC of 0.93 in identifying left ventricular (LV) systolic dysfunction in an unselected, large population setting. In [17], a deep learning model (DeepECG-HFrEF) was validated in symptomatic HF patients with EF < 40% and achieved an AUROC of 0.844. Regarding HFrEF vs. HFpEF classification, ref. [18] developed and evaluated traditional machine learning models; however evaluation was conducted on a single training/test split, limiting the assessment of the stability of the results. Another study [19], employed interval-based features (QTc, QRS, ST-T, and TP) extracted from 24 h Holter recordings and classified HFrEF, HFmrEF, and HFpEF (303 patients) using machine learning models, achieving AUROCs above 0.98. A study with the same cohort and a group of authors from the latter approached HF stratification by encoding 24 h circadian HRV features and clinical information into multi-parameter polar images, achieving an AUROC of 0.833 [20]. Additionally, using data from the MUSIC database and LightGBM, along with P-wave, QRS-complex and T-wave energies, ref. [21] achieved an accuracy of 98.45% and AUROC of 0.9989 for HFrEF vs. HFmrEF vs. HFpEF classification.

Regarding heterogeneity beyond EF subtypes, in [22], a transformer-based approach on large-scale electronic health records identified seven distinct HF subtypes, including unrecognized high-risk clusters, underscoring the heterogeneity of HF.

Beyond HF detection, wavelet-based ECG features combined with traditional machine learning models have also been explored for early-stage subclinical LV dysfunction [23,24,25,26], a condition related to HF pathophysiology, providing model interpretability through features suitable for specific physiological problems.

These works illustrate the variety of signal modalities and detection approaches applied to HF detection. Similarly, a meta-analysis of AI models for HF assessment showed that studies using CNN and larger datasets achieved consistently higher accuracy, while traditional machine learning models showed greater variability in their performance [27]. However, a common limitation persists. Vectorcardiography (VCG) provides a three-dimensional representation of cardiac electrical activity, which may reveal more detailed information than the standard 12-lead ECG [28]. In the cited studies, models based on deep learning methods and 12-lead ECG tend to offer limited interpretability. This deficiency does not allow clinicians to examine the features driving the classification, which is a critical requirement for individualized patient assessment and personalized care. Among the traditional machine learning approaches, ref. [18] relied on a single bipolar limb lead (I) without a healthy control group, while [19] used long-duration ambulatory recordings from a pseudo-orthogonal lead configuration, also without healthy individuals. All things considered, there is a motivation to develop interpretable, VCG-based approaches that incorporate features with physiological meaning, morphology and subtle rhythmic changes.

This study aimed to develop and internally evaluate a non-invasive ECG/VCG-based machine learning approach for HF assessment in individuals with sinus rhythm, with particular emphasis on HFpEF. The first endpoint was to identify patients with HFpEF from healthy individuals, and the second was to screen healthy individuals and patients with HFrEF and HFpEF. The model is intended as a potential screening tool to identify individuals who may benefit from further echocardiographic and biomarker-based evaluations rather than as a standalone diagnostic test.

## 2. Materials and Methods

### 2.1. Data Description

For this study, data were collected from two open-access databases from PhysioNet [29]: the PTB Diagnostic ECG Database [30,31] and MUSIC (Sudden Cardiac Death in Chronic Heart Failure) [32,33]. The PTB database is a collection of 549 high-resolution records (1000 Hz) from 290 subjects with different pathological conditions (some subjects have more than one recording). Each record contains 12-lead ECG, together with VCG leads in resting state, with a duration of 120 s. The MUSIC database contains 936 Holter ECGs and 687 high-resolution, resting-state VCG recordings sampled at 1000 Hz with a duration of up to 20 min (mean ± std), along with demographic, clinical and echocardiography data (EF) from ambulatory patients with chronic heart failure (CHF). From the PTB database, only healthy controls were included, resulting in 80 records (52 subjects); these subjects are hereafter referred to as the *normal* class. From the MUSIC database, 687 high-resolution VCGs were included at first, then filtered to exclude patients with ventricular tachycardia (VT), atrial fibrillation (AF), atrial flutter, bradycardia, prior implantable devices, pacemakers, and paroxysmal supraventricular tachyarrhythmia, resulting in 197 recordings (197 subjects) that were dominated by sinus rhythm (SR). This means that some micro-changes in rhythm might still exist (e.g., premature ventricular contractions—PVCs) and will be addressed in signal preprocessing. Distribution of samples among HF subtypes was HFrEF = 130, HFpEF = 50 and HFmrEF = 17. On account of the huge class imbalance and the need to diagnose and screen mostly patients with HFpEF, the HFmrEF class was incorporated into the HFrEF class, resulting in two HF classes: HFpEF (EF ≥ 50%) and HFrEF (EF < 50%). The EF thresholds were selected according to [34]. Table 1 presents the mean age and standard deviation, as well as the sex percentage per class. Although the MUSIC database provides more demographic information, the PTB database is limited only to age and sex, and some recordings have missing (NaN) values for these fields; the variable *n* indicates the number of samples of which this information was available.

### 2.2. Signal Preprocessing Pipeline

For the purposes of this study, a modular preprocessing pipeline was implemented with steps that could be applied selectively. Most of these steps were mandatory for the signal preprocessing pipeline for all the recordings. The optional steps were used exclusively because the signals from the two databases differed in quality and duration. Section 2.2.1, Section 2.2.2, Section 2.2.3, Section 2.2.4, Section 2.2.5, Section 2.2.6, Section 2.2.7, Section 2.2.8, Section 2.2.9 and Section 2.2.10 describe each preprocessing step (either mandatory or optional), and both Section 2.2.11 and Section 2.2.12 describe which of these steps and configurations were used for the MUSIC database and PTB database respectively.

#### 2.2.1. Edge Trimming

To prevent unwanted phenomena from the start and end of the signal, edge trimming was implemented by removing fixed seconds from the start and end of the signal. There is an option to adaptively trim the signal by inspecting the lead with the worst Median Absolute Deviation (MAD) for a given number of seconds, then trim the signal up to a given number of seconds.

#### 2.2.2. Detrend and High-Pass Filtering

To remove linear trend, baseline wander, and low-frequency noise artifacts, linear detrend and high-pass filter functions were implemented using an IIR Butterworth filter [35] with a cut-off frequency of 0.5 Hz and an order of 2.

#### 2.2.3. Clipping

In cases of high-amplitude noise due to electrode failure or motion artifacts, signal clipping at specific percentiles is crucial to avoid large artifacts that may affect R detection algorithms due to large differences in scale. For this analysis, the signals were clipped at the 0.01st and 99.99th percentiles to reduce artifacts while preserving the morphology of R peaks.

#### 2.2.4. Notch Filter

An IIR notch filter [36] was used to remove the power-line interference. In this study, 50 Hz and harmonics (100, 150, and 200 Hz) were suppressed because the recordings from the two databases were performed in Europe.

#### 2.2.5. Wavelet Filter

Biosignals, such as ECG, may contain noise that is specifically located in time and that a traditional filtering method might miss. Thus, a wavelet filter was implemented using Discrete Wavelet Transform (DWT) [37,38,39], which utilizes sym8 as the mother wavelet [40] and decomposes the signal to three levels (500–250 Hz, 250–125 Hz, and 125–62.5 Hz). This is a choice to avoid over-smoothing QRS complexes and retain most of the information. For each level, a different threshold was calculated using VisuShrink and soft thresholding [41].

#### 2.2.6. Bandpass Filter

To exclude any unwanted phenomena, a frequency range of 0.8 to 200 Hz was selected, and a Butterworth bandpass filter of order 10 (second-order sections) was applied.

**Figure A1:**
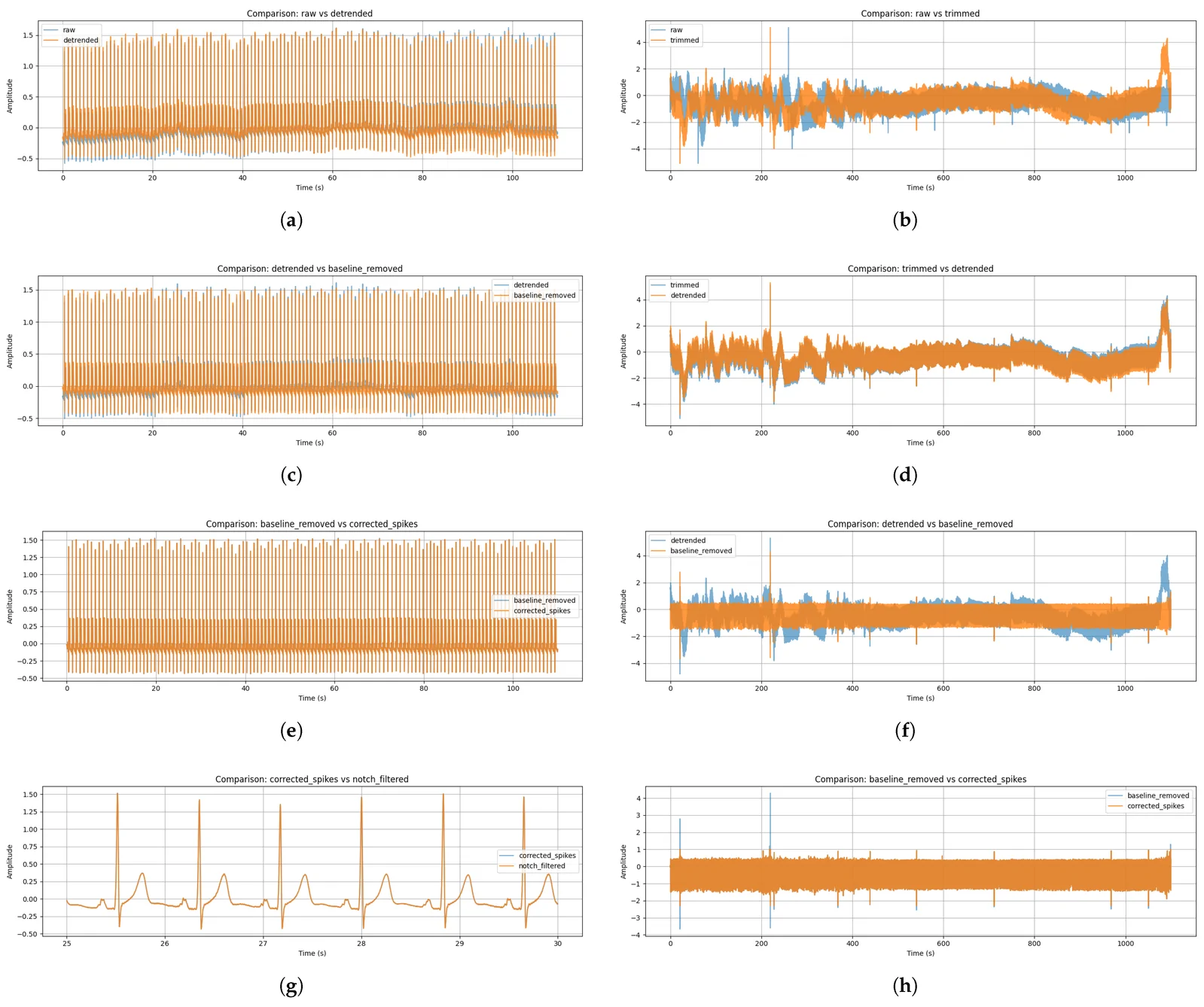

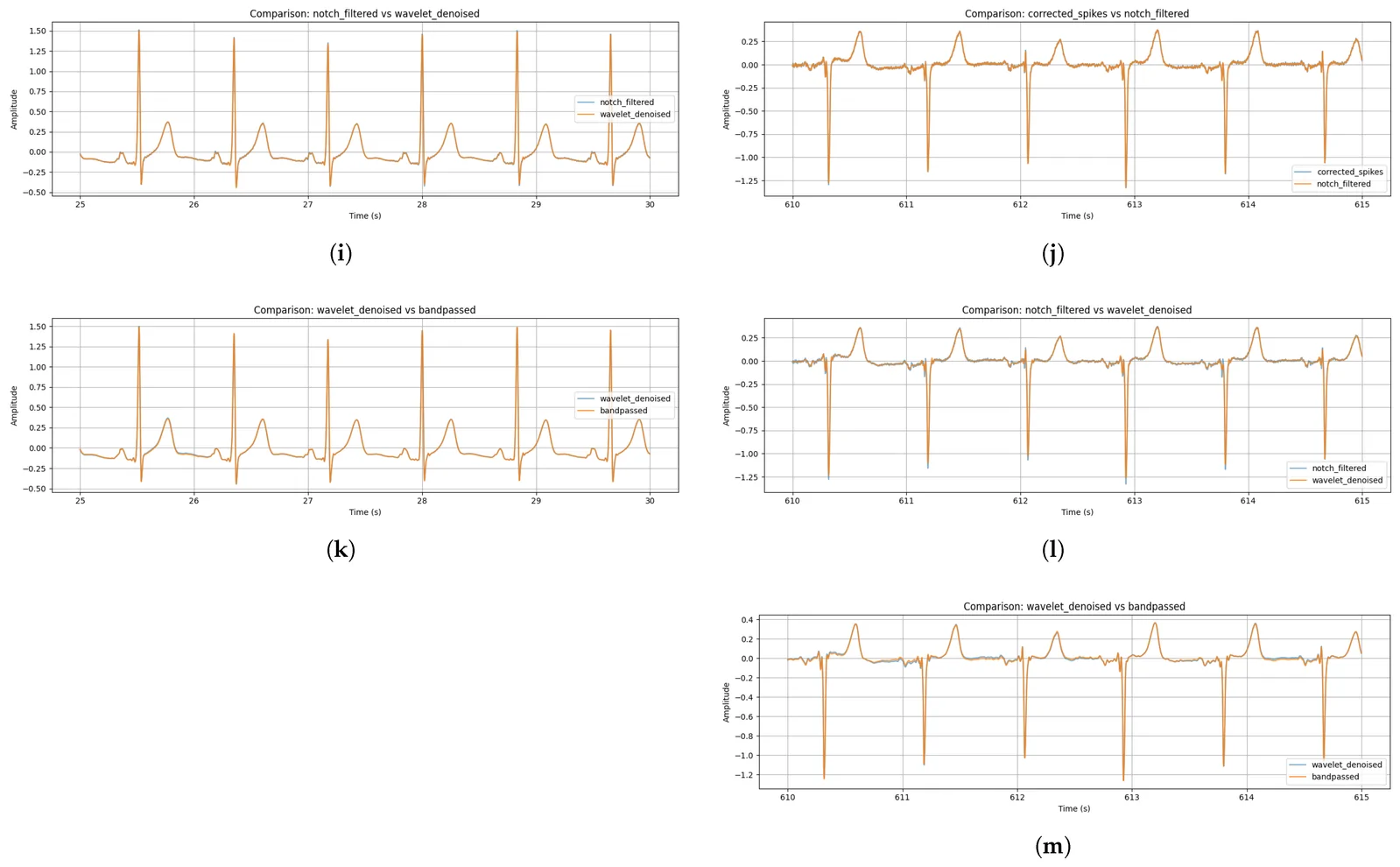
Denoising process step by step on a PTB signal (**left**) and a MUSIC signal (**right**). (**a**) raw vs. detrended signal (PTB); (**b**) raw vs. trimmed signal (MUSIC); (**c**) detrended vs. baseline removed signal (PTB); (**d**) trimmed vs. detrended signal (MUSIC); (**e**) baselined removed vs. spike-corrected signal (PTB); (**f**) detrended signal vs. baseline removed signal (MUSIC); (**g**) spike-corrected vs. notch filtered signal (PTB); (**h**) baselined removed vs. spike-corrected signal (MUSIC); (**i**) notch filtered vs. wavelet denoised signal (PTB); (**j**) spike-corrected vs. notch filtered signal (MUSIC); (**k**) wavelet denoised vs. bandpass filtered signal (PTB); (**l**) notch filtered vs. wavelet denoised signal (MUSIC); (**m**) wavelet denoised vs. bandpass filtered (MUSIC).

**Figure A2:**
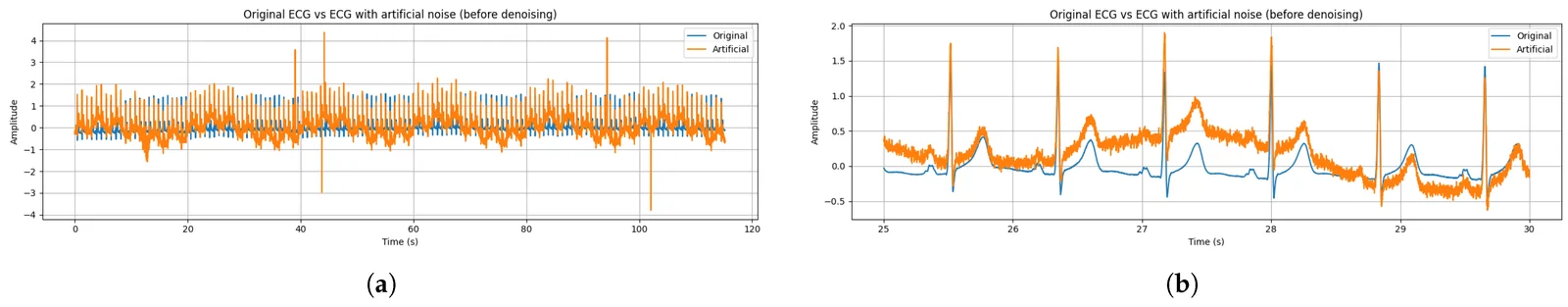
Comparison of denoising pipeline on an original raw signal and a copy of it with added artificial noise—before denoising. (**a**) Whole signal; (**b**) Segment of signal (25–30 s).

**Figure A3:**
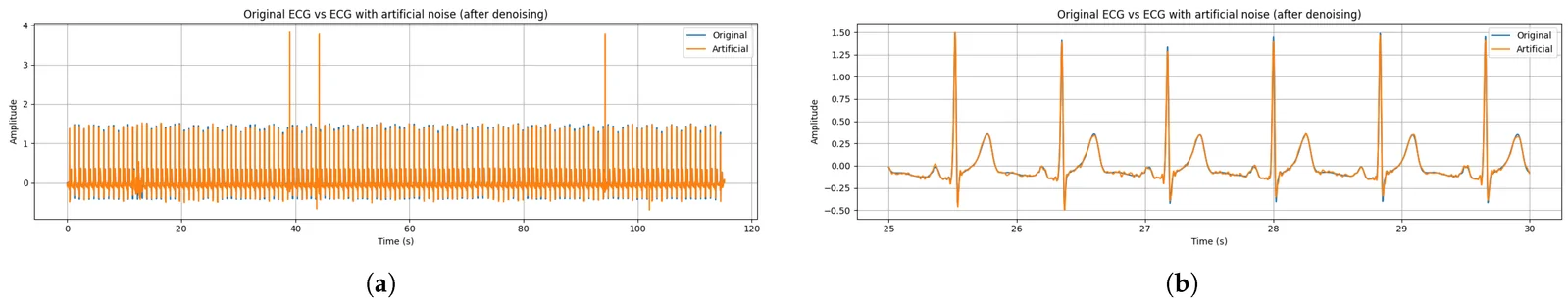
Comparison of denoising pipeline on an original raw signal and a copy of it with added artificial noise—after denoising. (**a**) Whole signal; (**b**) Segment of signal (25–30 s).

Each step of the denoising is depicted in Figure A1, and a comparison between an original signal vs. a copy of it with added artificial noise is presented in Figure A2 and Figure A3 in Appendix A.

#### 2.2.7. R Detection

To detect R peaks, XQRS and GQRS, together with the *correct_peaks()* algorithm, were used from the WFDB library [42]. First, XQRS is deployed, and the algorithm falls back to GQRS if the detected beats for each lead are less than expected or if the beats detected across leads (for the same recording) are more than expected. These constraints were approximated based on the signal duration and assuming that the ECG rhythm was SR. The mean heart rate (HR) for SR is 60–100 bpm for normal people. If the subject is an athlete, 40–100 bpm could be acceptable. While 40–100 bpm stands for the mean HR span, a 35–120 bpm span was selected for HR values that correspond to the minimum and maximum HR across the whole signal. To mitigate unwanted effects (R peak scoring that is very close to another), the detected R peaks were sanitized by excluding R peaks at the same indices and R peaks that were closer than 150 ms, an interval in the neighborhood of the scored QRS.

#### 2.2.8. Outlier Detection

**Figure A5:**
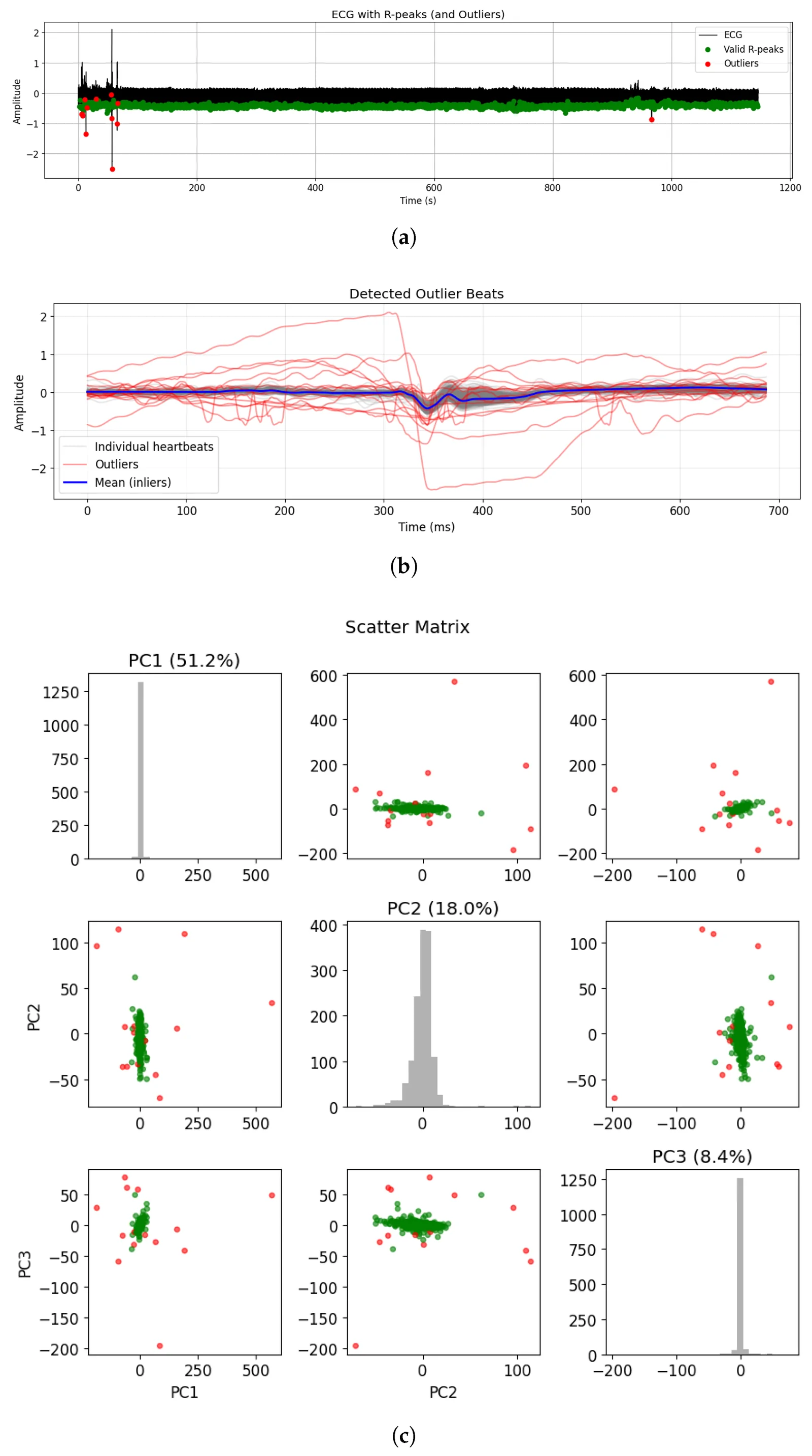
Outlier detection evaluation on a contaminated signal. (**a**) Visualization of valid R peaks and outliers across the whole signal; (**b**) All extracted heartbeats plotted together—each gray line represents each heartbeat, each red line an outlier beat, and the blue line the average heartbeat; (**c**) Scatter matrix of PC1, PC2 and PC3. Diagonal panels show the distribution (histogram) of each PC and off-diagonal panels show pairwise scatter plots with outliers marked in red.

Artifacts scored as R peaks or the presence of premature ventricular contractions (PVCs) in the signal can introduce bias into the models. To avoid this, principal component analysis (PCA) was deployed to determine outlier beats. This procedure begins by building a fixed-length window for all beats per lead, centered on each R peak using an adaptive window length based on RR intervals, with a minimum of 500 ms, since the upper limit is 120 bpm. To detect morphological changes and not differences in amplitude, each beat was normalized by subtracting the mean and dividing by the standard deviation. PCA was then applied using the Kaiser criterion [43] and setting the minimum and maximum number of components to 3 and 20, respectively. To detect which beat was an outlier, the PCA data were first scaled to have a mean of 0 and a standard deviation of 1 to isolate the segment’s shape, regardless of its amplitude. Next, a nearest-neighbor model (kNN) [44,45] was fitted with k equal to the number of PCA components plus one (rule of thumb). Using the distances from the kNN model, KneeLocator [46] was utilized to find the optimal epsilon to fit the DBSCAN [47] value, with the sensitivity (S) set to 1.0 for an increasing convex curve [48,49]. For the kNN model and DBSCAN, the outliers were found using the cosine metric, which was selected based on the best performance. This resulted in a mask with outlier beats marked as Boolean false and valid beats as Boolean true. Outlier detection examples—one from a clean signal and one from an artifact-contaminated signal—are presented in Appendix A.2 and Figure A5 in Appendix A.

#### 2.2.9. Best Window Selection

Recordings in the databases had different durations; therefore, a fixed-length window was required to compute comparable time-domain (HRV) features across recordings. A sliding window of 120 s, moving in steps of 10 s across each signal, was used and automatically selected the signal segment that minimized a score combining the number of outliers within the window and a standard deviation (std) term based on the median absolute deviation (MAD) of the worst lead:

(1) score=numberofoutliersinsidetheslidingwindow+MAD-basedstdterm

Across the three leads, the same start and end index was applied for a given recording. After determining the optimal signal segment, the R-peak indices were re-referenced to a local time frame (0–120 s) by subtracting the window’s global start index from each R-peak’s global index. This produced one 120 s ECG segment per recording, which was used for the feature extraction step.

#### 2.2.10. Signal Standardization

ECG signals from two different databases were acquired using different devices. To detect differences in morphology and not in signal scales, every lead in every recording was divided by its standard deviation.

#### 2.2.11. MUSIC Database Configuration

Recordings from the MUSIC database were trimmed to a fixed value of 10 s from the start and the end and adaptively trimmed, looking at 60 s to calculate the MAD, and trimmed up to 30 s more. The maximum difference that was accepted in between leads before using the GQRS algorithm was 50 beats, and the minimum number of R peaks for every lead was 400 because most of the recordings had a duration of 10 to 20 min (most of them had a 20 min duration). ECG segmentation was applied as mentioned in Section 2.2.9 to make equal-length ECGs with the PTB database.

#### 2.2.12. PTB Database Configuration

Recordings from the PTB database were not fixed-trimmed or adaptively trimmed. The inter-lead agreement threshold for falling back to the GQRS algorithm was set to 3 beats, and a minimum number of 30 R peaks per lead was required. All recordings were already 120 s in duration; thus, no segmentation was applied.

### 2.3. Feature Extraction

HRV features were computed to detect micro-changes in rhythm (patients with SR) and CWT features to detect differences in frequency components of the heartbeats, resulting in major or subtle morphological/spectral changes. To mitigate any artifacts that were not detected from PCA, a “safety lock” was deployed by keeping RR intervals strictly between 500 and 1700 ms (35–120 bpm). Outliers masked as false elements by the PCA mask were not involved during the feature calculation process.

#### 2.3.1. HRV Features

It was observed that lead VX had the fewest outliers during experimentation with signals; therefore, it was used to calculate the set of features [50] shown in Table 2.

#### 2.3.2. CWT Features

For this analysis, CWT with the real Morlet wavelet was applied [51,52] to the QRS and ST-T waves for each beat, and several features were computed. To calculate the wavelet scales, a target frequency band was first chosen, and a dense geometric frequency grid was constructed (256 points), with each frequency converted to the corresponding scale. Each beat was defined in an adaptive window using half of the RR interval for each pair of R peaks. The QRS complex was defined as a fixed window of 120 ms (±60 ms around the R peak), and an area containing the ST interval and T wave as a window of ≤300 ms, immediately after the QRS complex. The QRS and ST-T features [53] were computed separately for different frequency bands, as shown in Table 3. For each beat and frequency band, the features listed in Table 4 were calculated and aggregated (mean and std). While different in scope relative to this work, the band selection was inspired by the work of [54]. The diagnostic ECG bandwidth has been proposed to have an upper cutoff of 150 Hz [55] or even 250 Hz [56]. The fundamental frequency of the QRS complex is approximately 10 Hz, and most of the diagnostic information is contained below 100 Hz [55]. The four bands cover this range, excluding 200–250 Hz for computational complexity and noise mitigation purposes. While the physiological T-wave frequency ranges from 0 to 10 Hz, the rationale for the ST-T band selection is that higher frequencies might appear in the disease. However, their range does not extent to very high frequencies in respect of the T wave’s slower nature. This is not an established frequency-band scheme and has not been validated in prior studies. It should be treated as exploratory. Further analysis to determine the optimal ranges could be explored, but it is not in the scope of the current study.

### 2.4. Machine Learning Pipeline

To examine the first and second endpoints, machine learning steps were implemented to achieve unbiased estimations, given the small size of the dataset. The architecture is based on binary classifications to examine how well a classifier distinguishes a pair of classes (normal vs. HFpEF, normal vs. HFrEF, and HFrEF vs. HFpEF) and, later, the aggregation of three models into an n^2^ classifier to examine the second endpoint.

#### 2.4.1. Feature Selection

For every pair of classes, an iterative/consensus feature selection approach was implemented to ensure that the set of features represented the entire dataset and avoided biased estimations from only one training set. The procedure begins with splitting of the new training and test sets. For the training set, the features that were not statistically significant (*p* > 0.05) using the Kruskal–Wallis test—as feature space did not follow a normal distribution—[57] and correlated (ρ ≥ 0.8) using the Pearson correlation method were dropped. For the remaining features scaled to have a mean of 0 and a standard deviation of 1, SMOTE [58] was used to augment the data and tackle any imbalances, and a RENT classifier [59] selected the most representative features. This followed for 100 iterations, and the feature set that was finally selected contained the features that were chosen in ≥50% of the iterations. For this threshold, the majority rule (50%) was considered to reach a consensus on the selected features. The inspiration for feature selection was the work of [60,61]. To assess whether features capture physiology-related changes rather than database origin, the batch effect was assessed (see Appendix A).

#### 2.4.2. Binary Classification

Similarly, binary classification was performed following a nested cross-validation variant for 100 independent train/test splits to increase the robustness of the models. For every iteration, the SVM [62], RF [63] and XGBoost [64,65] models are trained by up-sampling the training data with SMOTE or not and using GridSearchCV (see hyperparameter ranges in Table A5 in Appendix B) and StratifiedKFold for 5 cross-validation splits, where folds were constructed by randomly shuffling the data prior to splitting to reduce ordering bias. The Matthews Correlation Coefficient (MCC) [66] was used as the scoring metric for hyperparameter tuning because it uses all four parts of the confusion matrix (CM) and is a more reliable statistical metric than the F1 score in imbalanced datasets [67]. Model performance on the test set was evaluated using accuracy, precision, recall, F1 score, Area Under the Receiver Operating Characteristic Curve (AUROC) score, specificity, balanced accuracy and MCC.

Using the results of all iterations, the mean values and confidence intervals (CIs) for every metric were calculated, and the best classifier was selected according to the best mean MCC value. For this model, the AUROC and CM were calculated for all iterations (for CIs, AUROC, and CM calculations for the 100 iterations, see the Appendix C).

The selection of the best classifier did not involve the selection of the best hyperpara meters—only the best-performing algorithm (SVM, RF, or XGBoost) and either the use of SMOTE or not were assessed. To find the best models and validate their performance internally, a final training was performed for the best classifier with hyperparameter tuning (as described above) in 3 different training sets of the whole dataset, accompanied by 3 corresponding test sets for every binary classification pair, resulting in 3 models per classification pair.

### 2.5. Patient Screening

The trained binary models were aggregated into an n^2^ classifier [68]. To achieve this, the entire test dataset was loaded, and sub-datasets were created that contained only the features selected for each model. For every sample, each model makes a prediction. and the prediction scores for every class are calculated as follows:

(2) $$\begin{array}{cc} score_{normal} & =\left(1-prediction_{normalvsHFrEF}\right)+\left(1-prediction_{normalvsHFpEF}\right) \end{array}$$

(3) $$\begin{array}{cc} score_{HFrEF} & =prediction_{normalvsHFrEF}+\left(1-prediction_{HFrEFvsHFpEF}\right) \end{array}$$

(4) $$\begin{array}{cc} score_{HFpEF} & =prediction_{normalvsHFpEF}+prediction_{HFrEFvsHFpEF} \end{array}$$

These scores are then fed into the softmax function [69], and the probability of a sample being included in each class is calculated. The class with the highest probability is considered as the final class prediction for the sample.

To evaluate the capability of the n^2^ classifier to screen subjects, the accuracy per class, AUROC, CM, and F1 score were calculated, together with the overall accuracy, macro recall, macro precision, and macro F1 score.

To investigate feature importance, the permutation feature importance (PFI) [70] method, complemented by SHAP analysis [71], was utilized on the model for each classification task that achieved the best performance among the three.

### 2.6. Sample Size and Power Analysis

Post hoc statistical power was evaluated for the binary classifiers and the combined n^2^ classifiers, given the observed class sizes. The power of binary classifiers was computed using the Hanley–McNeil method, linking AUROC to its sampling variance [72,73], and the n^2^ classifier power was computed as a one-sided binomial test of overall accuracy against the no-information rate (NIR). The precision of class-specific performance was assessed using 95% Wilson-score confidence intervals, and model stability was checked with events-per-variable (EPV) ratios [74]. The formulas and tables are provided in Appendix D.

## 3. Results

### 3.1. Binary Classifications

**Figure 1:**
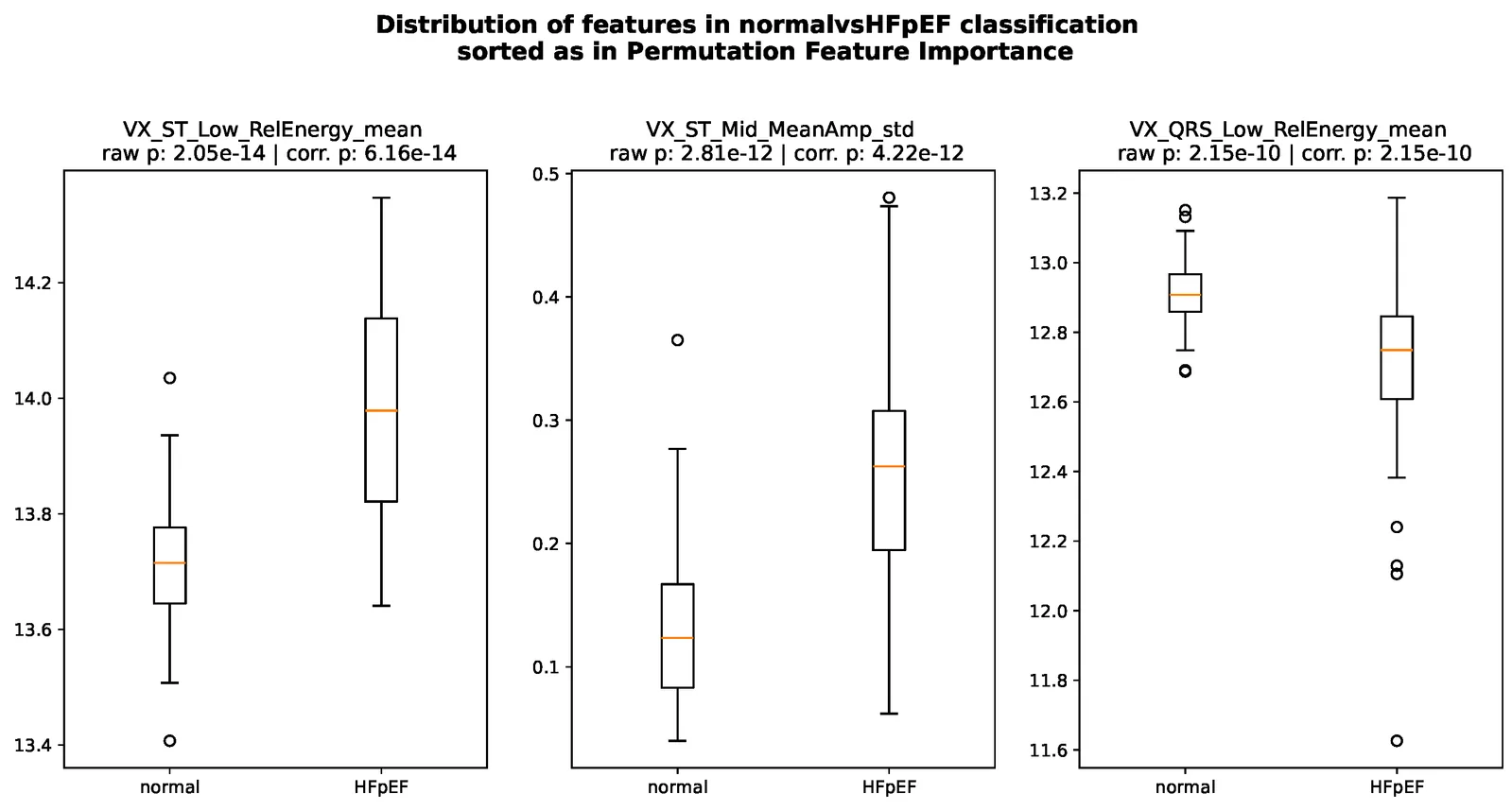
Feature distribution with raw and corrected *p*-values (FDR) from the KW test—normal vs. HFpEF dataset; the orange line represents the median while the circles represent outliers.

**Figure 2:**
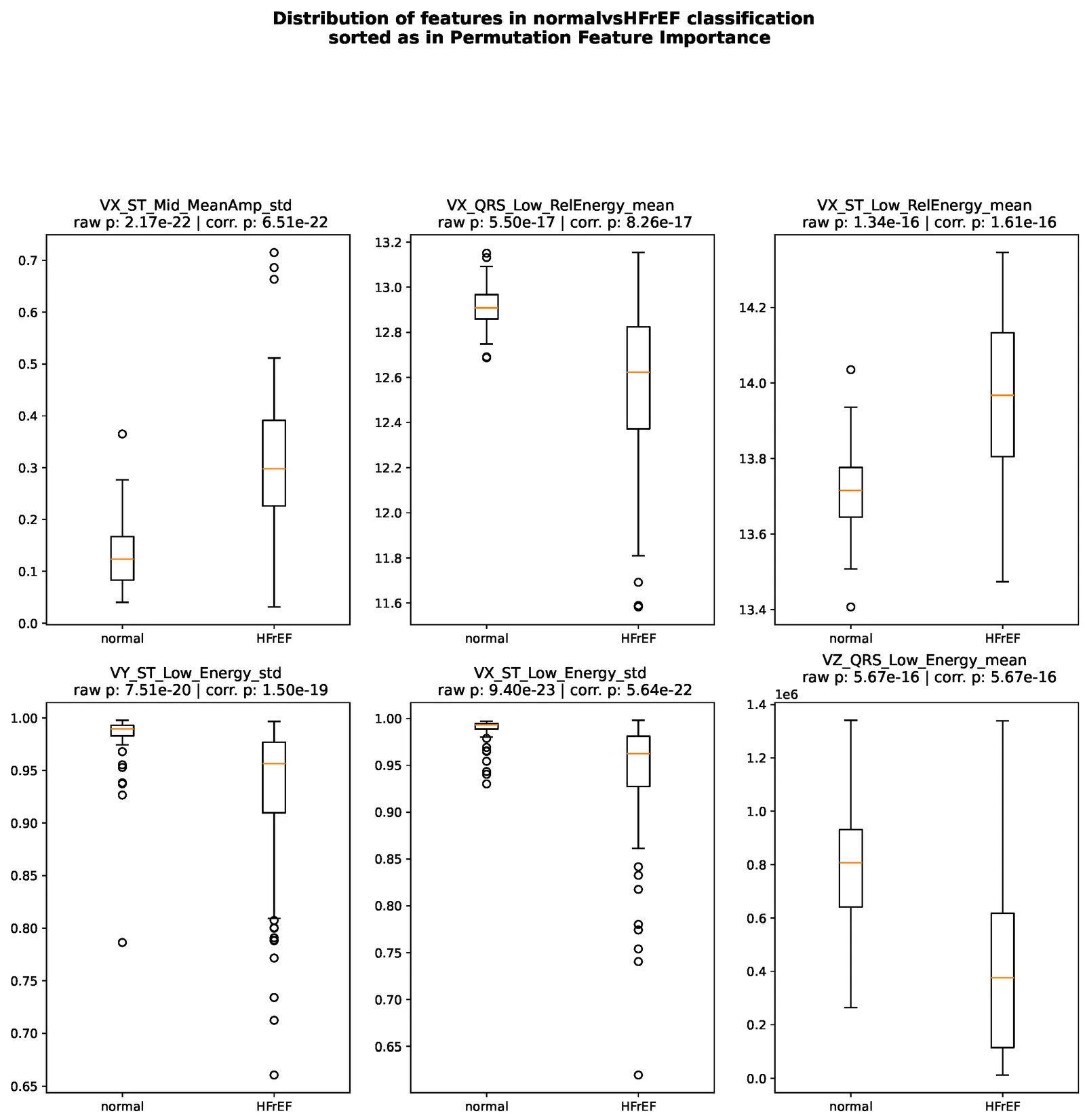
Feature distribution with raw and corrected *p*-values (FDR) from the KW test—normal vs. HFrEF dataset; the orange line represents the median while the circles represent outliers.

**Figure 3:**
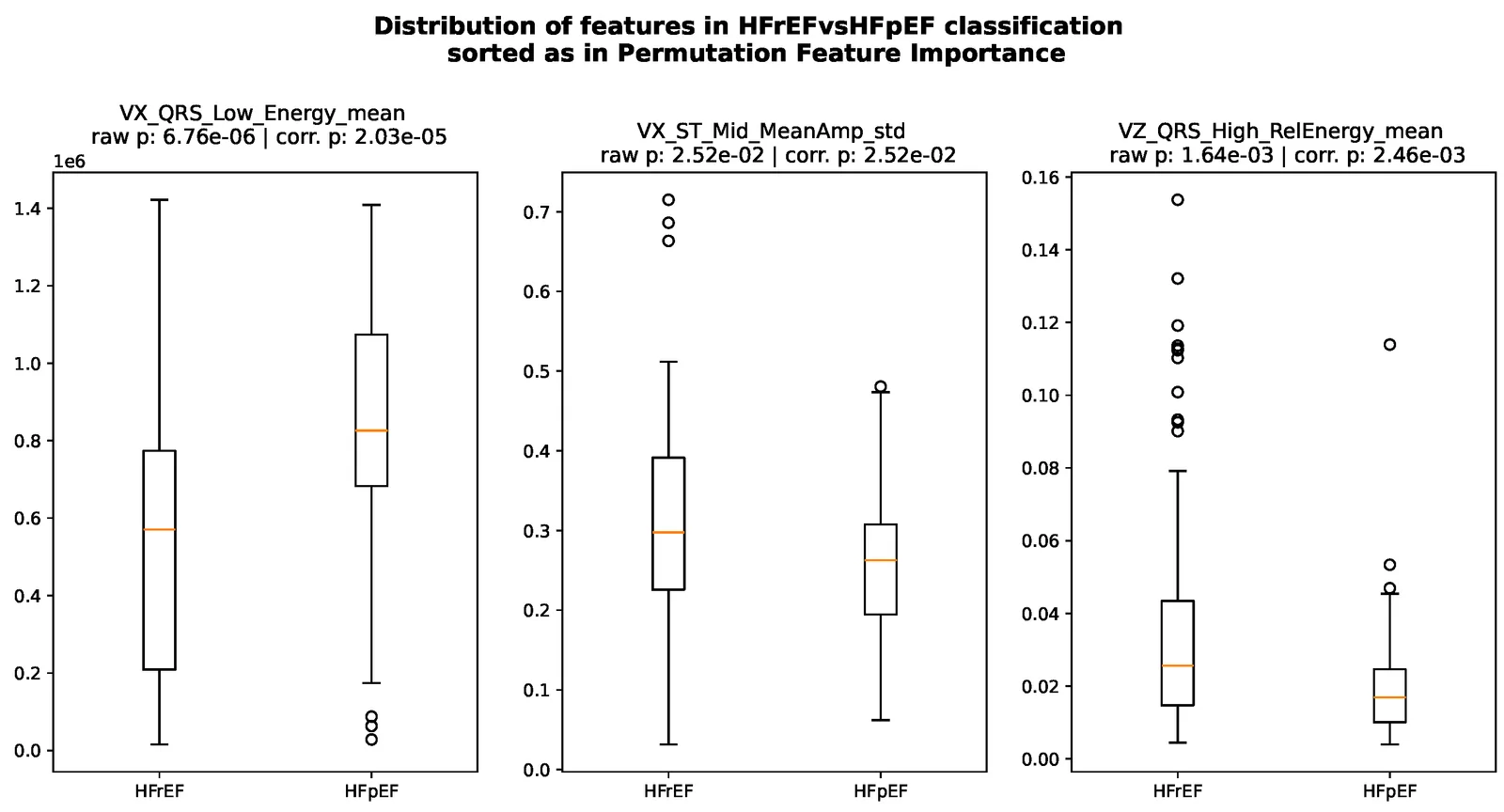
Feature distribution with raw and corrected *p*-values (FDR) from the KW test—HFrEF vs. HFpEF dataset; the orange line represents the median while the circles represent outliers.

From the feature selection step, RENT identified three features as the most significant for the normal vs. HFpEF binary classification. Regarding the normal vs. HFrEF and HFrEF vs. HFpEF binary classifications, RENT also identified the best features to be utilized for the second endpoint. All selected features are listed in Table 5, and their distributions are shown in Figure 1, Figure 2 and Figure 3. The results from batch effect assessment are presented in Appendix A.3.

**Figure 4:**
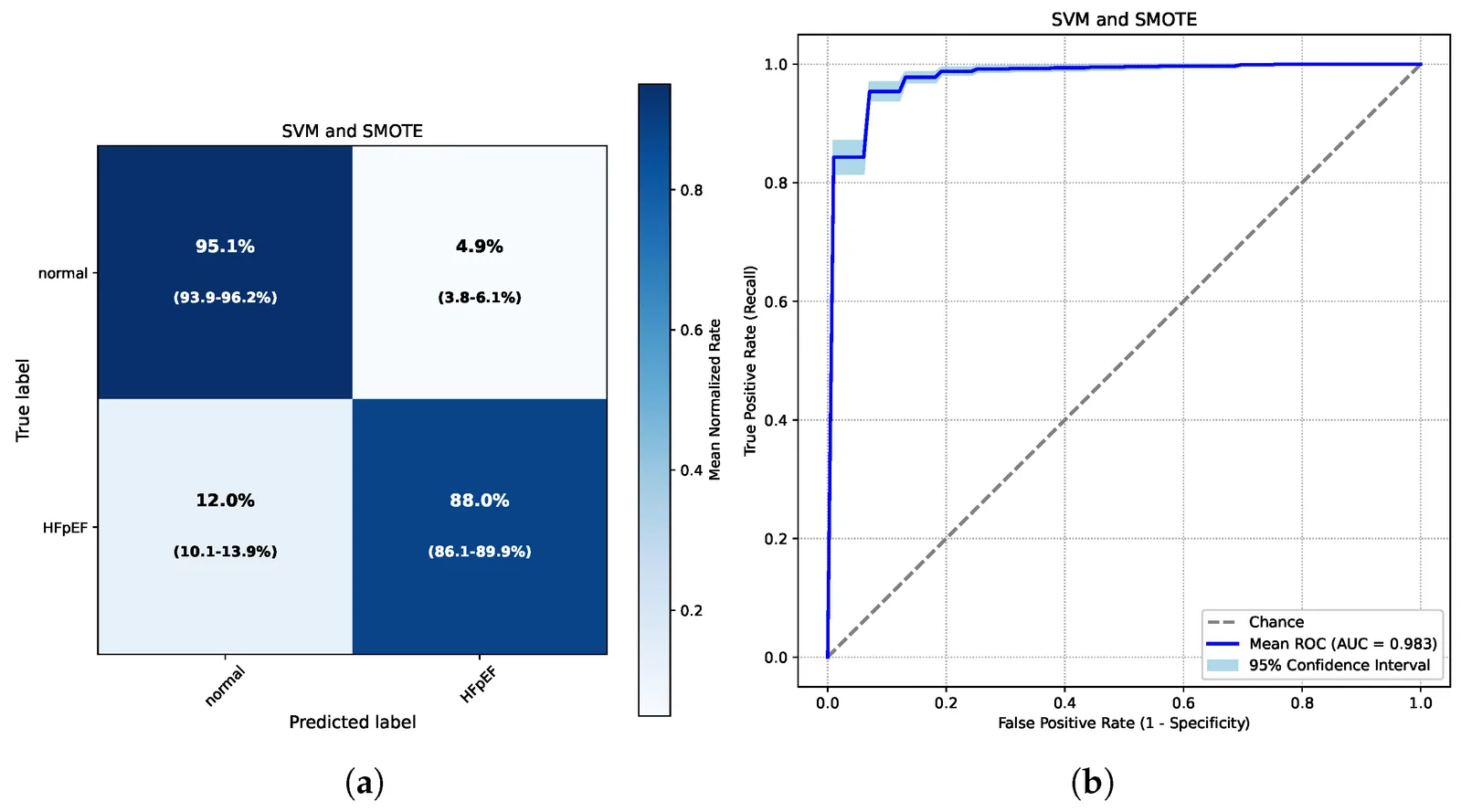
Normal vs. HFpEF model results for 100 classification repetitions. (**a**) confusion matrix; (**b**) AUROC curve.

Regarding the binary classification results, the best classifier for normal vs. HFpEF was the SVM using SMOTE. The metrics for 100 different training and test sets, along with their confidence intervals, are presented in Table 6. The confusion matrix and AUROC curve are shown in Figure 4.

**Figure 5:**
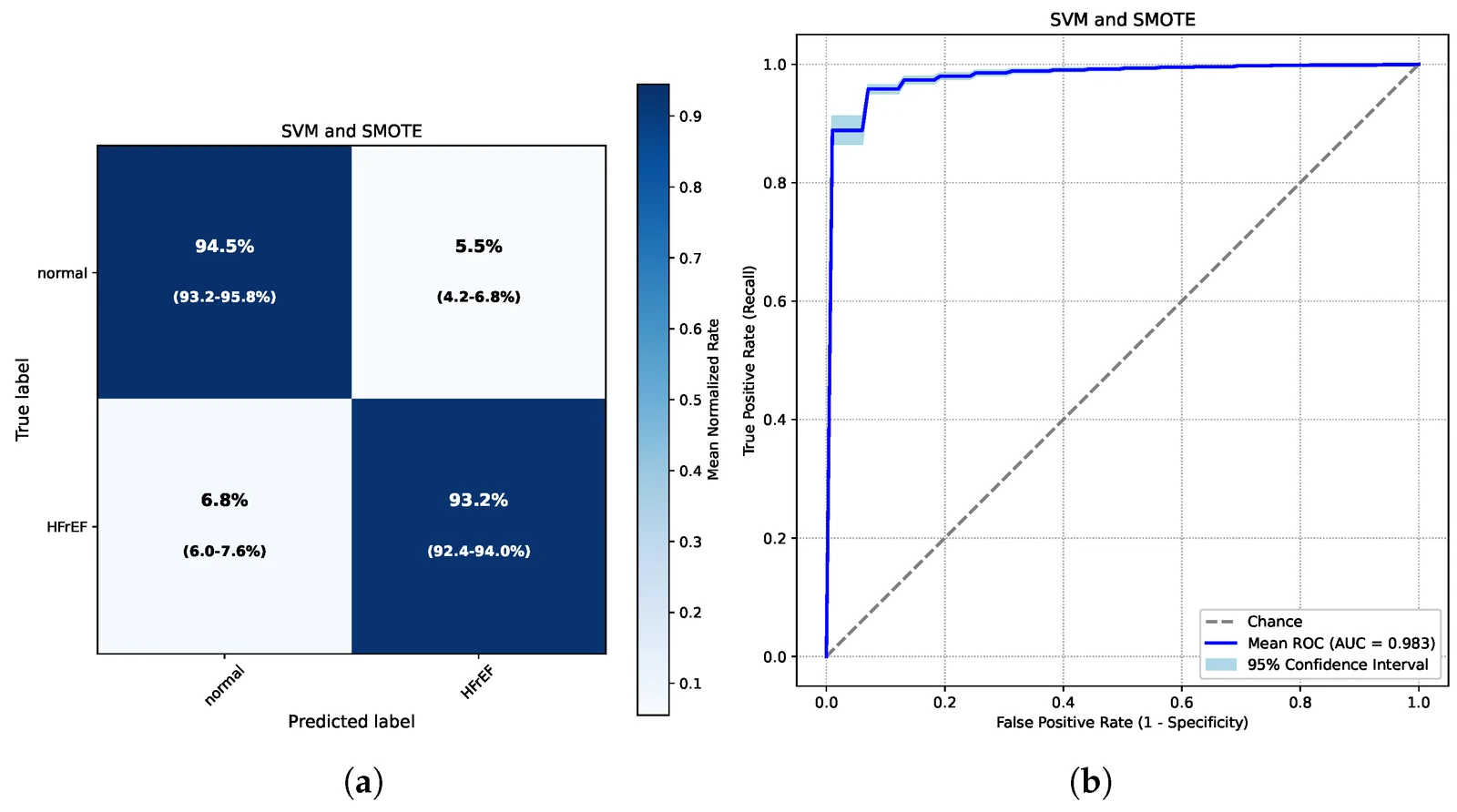
Normal vs. HFrEF model results for 100 classification repetitions. (**a**) confusion matrix; (**b**) AUROC curve.

**Figure 6:**
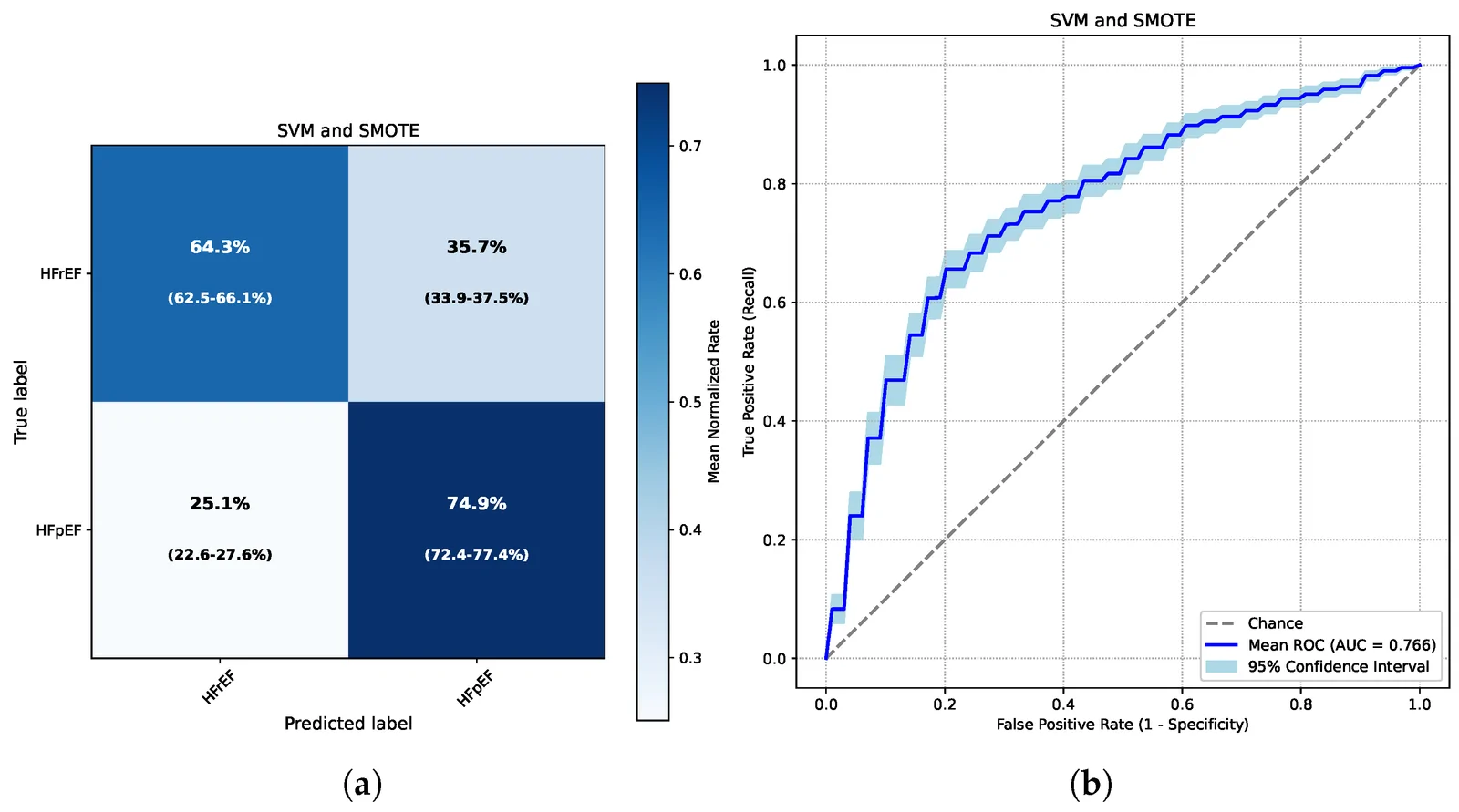
HFrEF vs. HFpEF model results for 100 classification repetitions. (**a**) confusion matrix; (**b**) AUROC curve.

The performance metrics, confusion matrices, and AUROC curves of the normal vs. HFrEF and HFrEF vs. HFpEF models are also presented in Table 7 and Figure 5 and Figure 6. The hyperparameters of the selected models are presented in Table A6 in Appendix B. Additional results of the models that were not selected as the primary ones are shown in Data S1, S2 and S3, and performance metrics for each iteration per classification task are presented in Data S4, S5 and S6 (see Supplementary Materials).

### 3.2. Subtype Stratification

**Figure 7:**
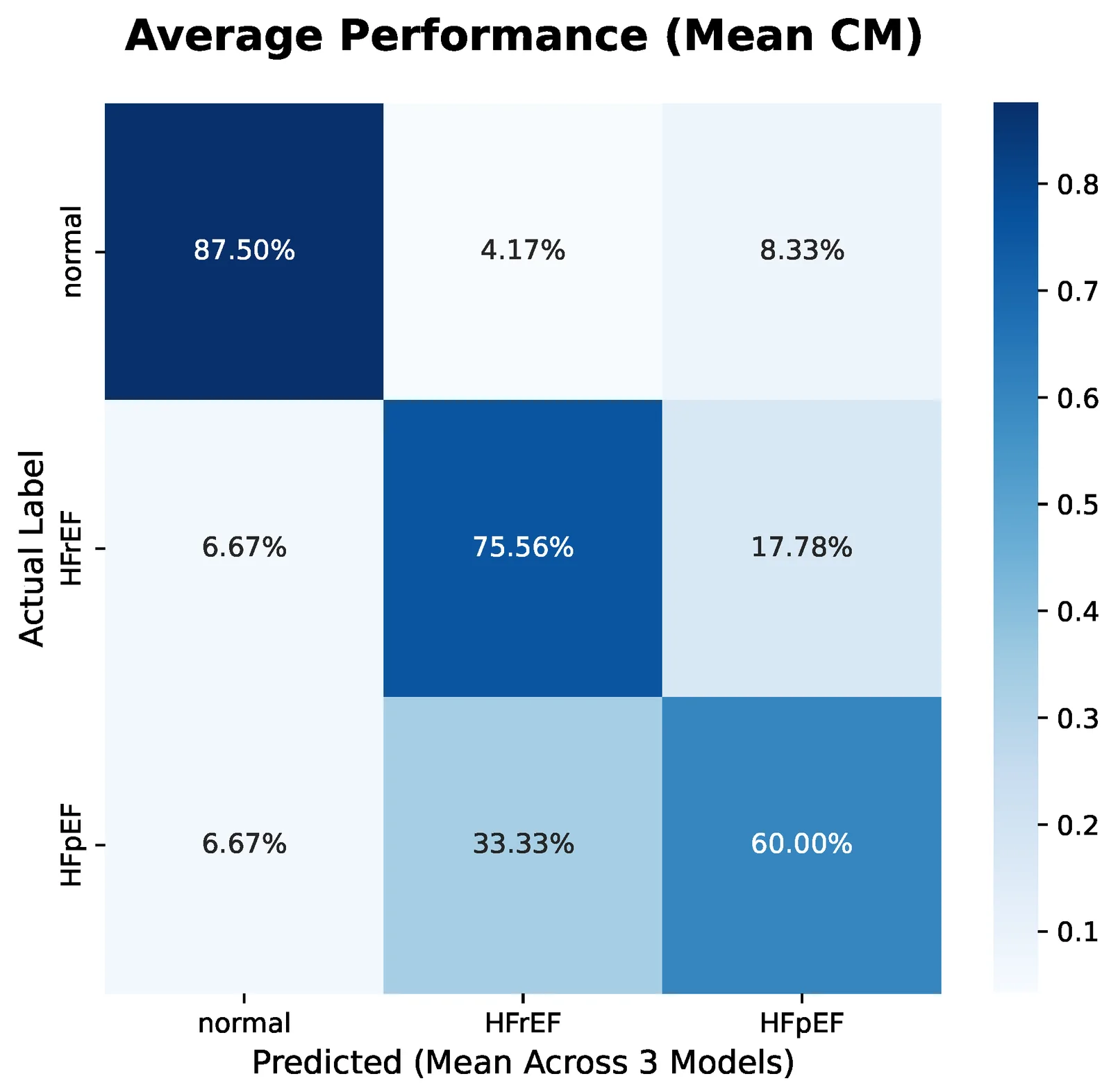
Mean confusion matrix from patient screening for 3 test sets.

**Figure 8:**
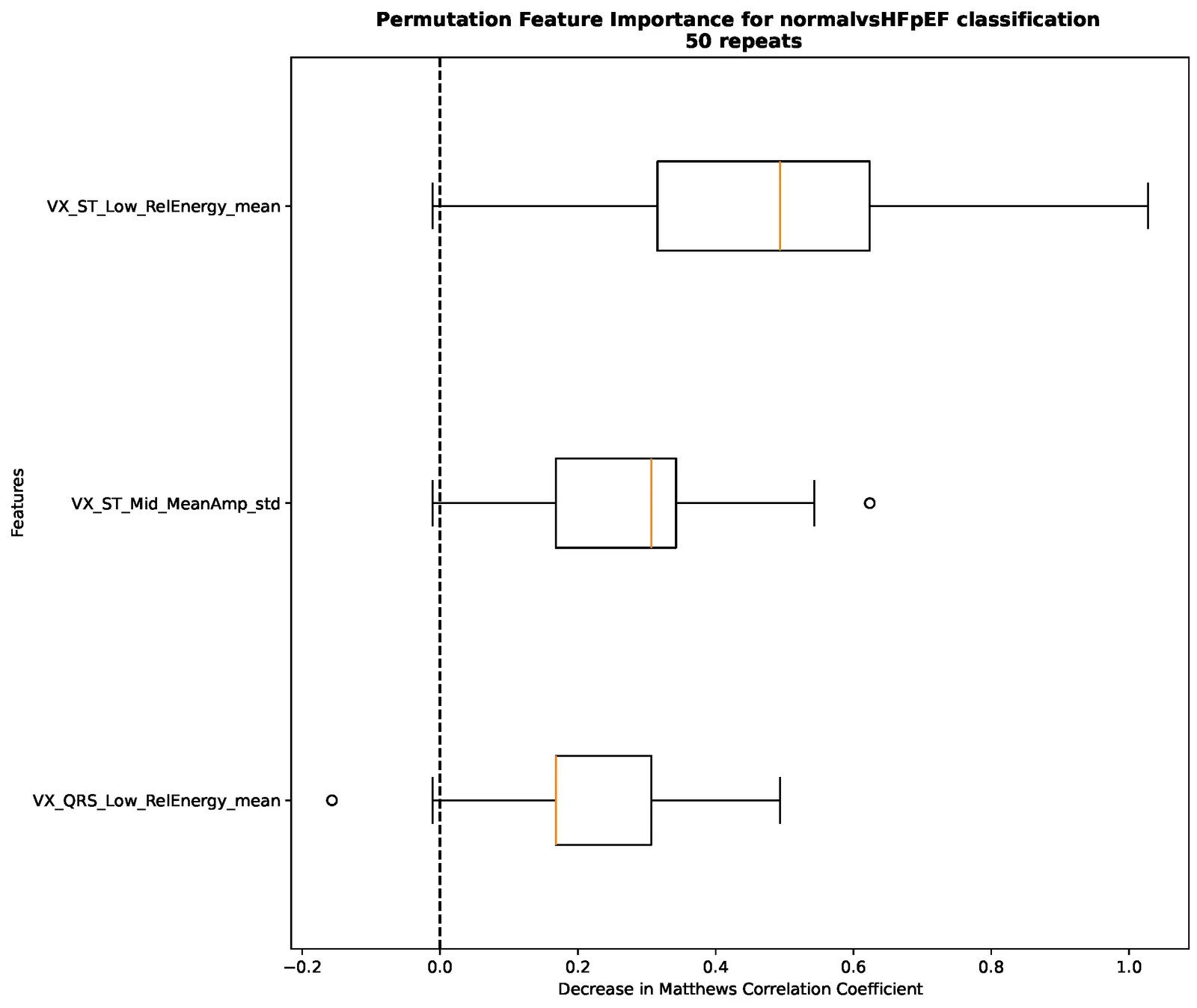
Permutation Feature importance—normal vs. HFpEF model (13 samples); the orange line represents the median while the circles represent outliers.

**Figure 9:**
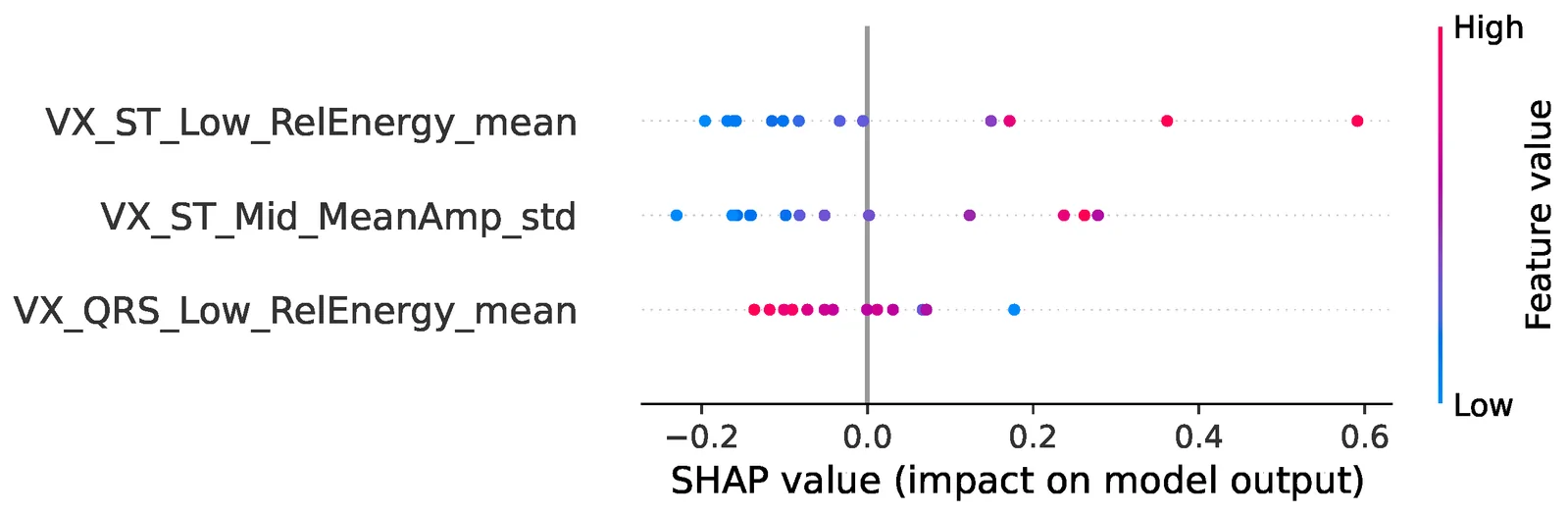
SHAP values—normal vs. HFpEF model (13 samples).

**Figure 10:**
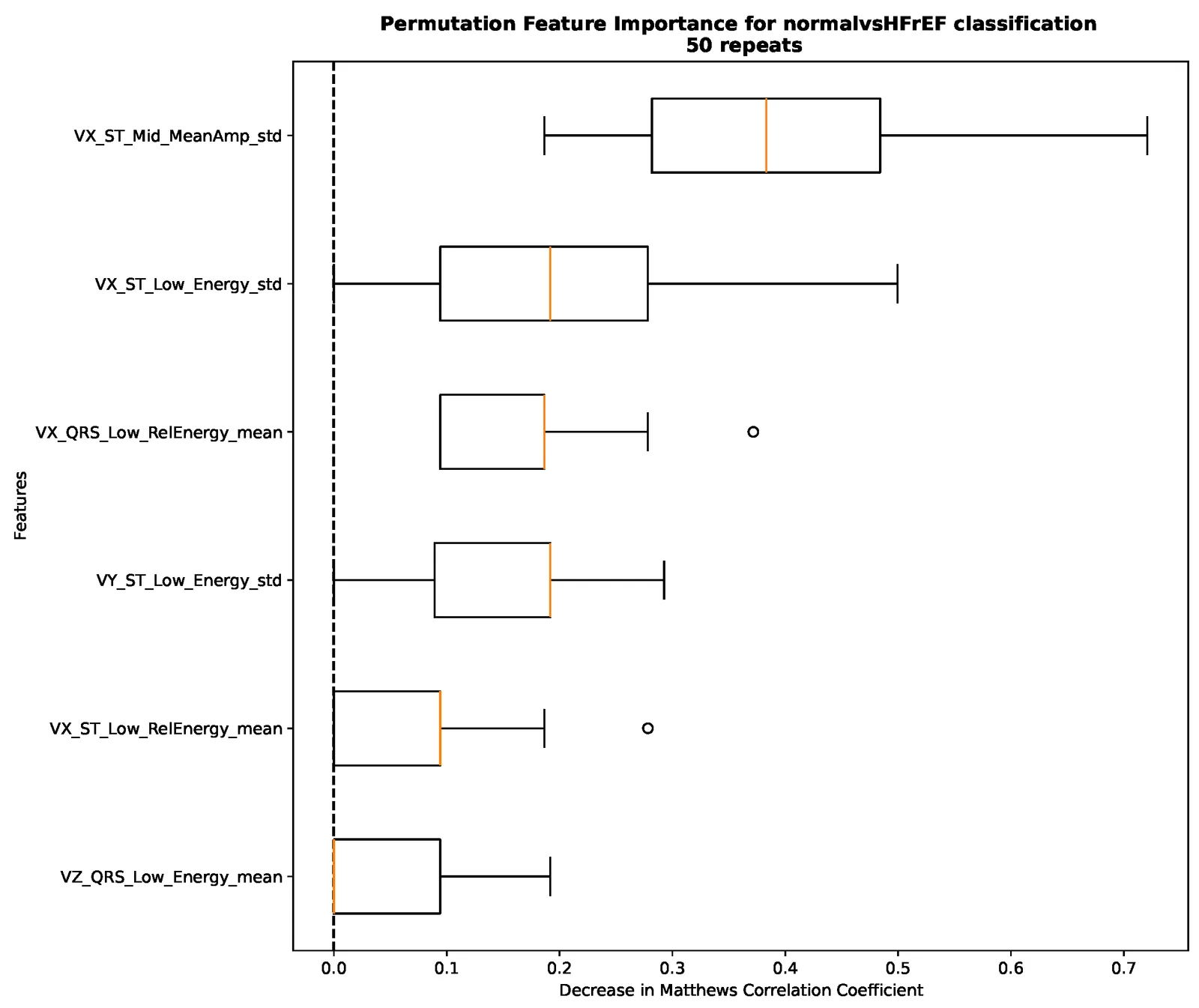
Permutation feature importance—normal vs. HFrEF model (23 samples); the orange line represents the median while the circles represent outliers.

**Figure 11:**
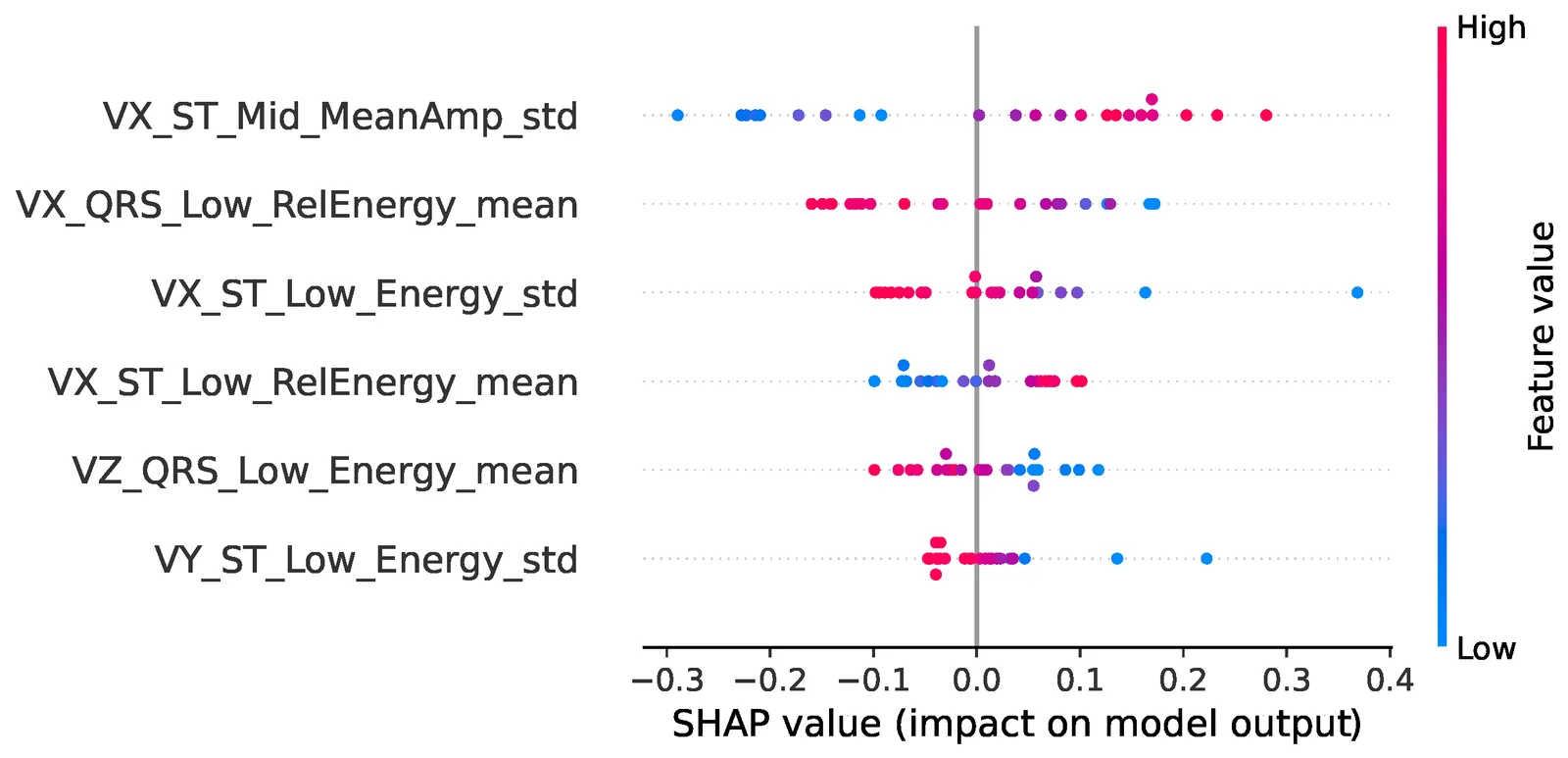
SHAP values—normal vs. HFrEF model (23 samples).

**Figure 12:**
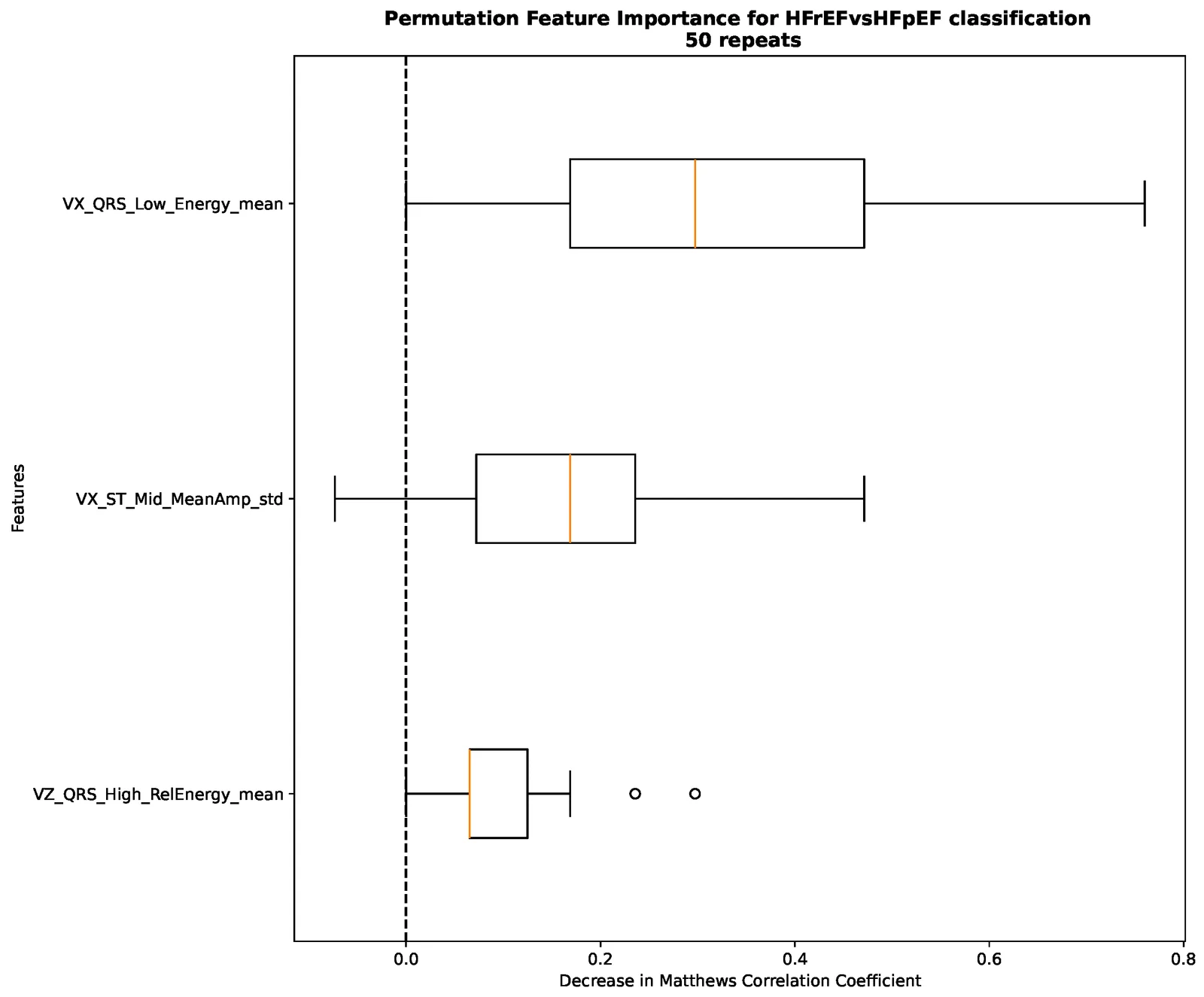
Permutation feature importance—HFrEF vs. HFpEF model (20 samples); the orange line represents the median while the circles represent outliers.

**Figure 13:**
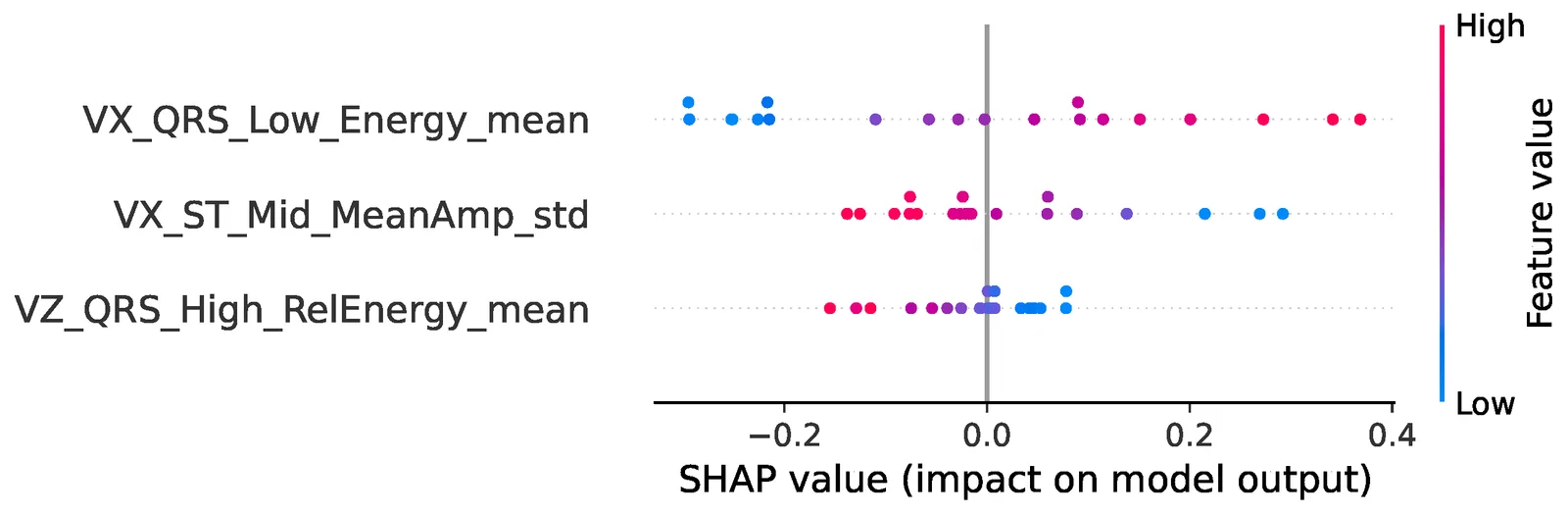
SHAP values—HFrEF vs. HFpEF model (20 samples).

Concerning patient screening, the results of the three test sets—three different n^2^ classifiers—were aggregated and are presented in Table 8, together with the mean confusion matrix in Figure 7. The best n^2^ classifiers were selected, and the PFI, together with SHAP values, were calculated for every classification pair, as presented in Figure 8, Figure 9, Figure 10, Figure 11, Figure 12 and Figure 13.

## 4. Discussion

### 4.1. The Value of This Work in Personalized Medicine

Heart failure management needs differ among patients and require interventions that focus on each patient individually. To create representative clusters of phenotypes, each HF subtype must be precisely diagnosed and stratified. This study demonstrated that ECG, a widely available clinical examination, has the potential to aid in HF assessment, with some considerations regarding HF subtype classification. In detail, normal vs. HFpEF and normal vs. HFrEF models exhibited impressive and robust performance for multiple train/test splits. This is evident from the metrics: Recall = 0.880 (95% CI: 0.861–0.899), F1 score = 0.897 (95% CI: 0.885–0.909), and AUROC = 0.983 (95% CI: 0.979–0.987) for the first one and Recall = 0.932 (95% CI: 0.924–0.940), F1 score = 0.950 (95% CI: 0.945–0.955), and AUROC = 0.983 (95% CI: 0.980–0.986) for the latter one. Although the best models included SMOTE use, Data S1 and S2 (see Supplementary Materials) show that performance metrics without SMOTE differ to a very limited extent.

The HFrEF vs. HFpEF model showed that it could identify most of the true positives by its metrics—Recall = 0.749 (95% CI: 0.724–0.774) and AUROC = 0.766 (95% CI: 0.749–0.782)—but was susceptible to false positives. The difficulty of discriminating between HFrEF and HFpEF is also shown in the aggregated results from the n^2^ classifiers. Data S3 (see Supplementary Materials) shows that without SMOTE, the models severely underperformed. It should be acknowledged that its use might produce a bias in the HFrEF vs. HFpEF model, and these findings should be interpreted cautiously due to the lack of an external validation set.

The findings suggest that the proposed approach may be more suitable as an HF screening tool, whereas EF subtype classification should currently be considered exploratory and requires validation in larger, well-phenotyped cohorts. However, most patients with HF are not undiagnosed, allowing clinicians to proceed with further tests to determine EF, identify the phenotype to which they belong and treat them accordingly.

### 4.2. Clinical Relevance of the Selected Features

HRV is consistently reduced in congestive HF, reflecting autonomic imbalance (increased sympathetic versus vagal tone), and it is generally related to cardiovascular risk [75], constituting a potential biomarker for continuous monitoring, and prognostic value (especially in terms of longitudinal variations of HRV features). While HRV features are relevant to HF, they were not selected, which is consistent with the controversial findings reported in the review by Míková et al. [76]. Although STD HR, SD1, SD2, NN50, pNN50, SDNN, RMSSD and Max HR were found to statistically significant between normal vs. HF and Max HR between HFrEF vs. HFpEF (see Data S4 in Supplementary Materials), their importance was not higher than the ECG morphology features. The ultra-short term window for HRV feature calculation may limit diagnostic value.

HFrEF is characterized by more severe conduction abnormalities, such as a wide QRS, left bundle branch block (LBBB), pathological Q waves, and left ventricular hypertrophy (LVH) [77], whereas HFpEF shows subtler changes in electrical activity, such as LVH with repolarization abnormalities, myocardial fibrosis, and diastolic dysfunction [78]. These underlying pathologies leave a footprint in the frequency domain. Within the QRS complex, the low-frequency content is influenced by ventricular conduction time and depolarization such that slow conduction and widening of QRS distribute power towards the lowest frequencies, where normal QRS already concentrates most of its spectral energy [79]. In contrast, QRS with high-frequency content is linked to fragmentation by fibrosis and microscarring [80]. In the repolarization interval, structural remodeling (LVH and myocardial fibrosis) increases repolarization variability, resulting in secondary changes in the ST segment and alterations in T-wave morphology, which reflect heterogeneous recovery of the myocardium [81,82,83].

These physiological associations were drawn from the broader literature on QRS and ST-T pathophysiology in heart disease. The CWT-based features identified in this study should be interpreted as hypothesis-generating electrical signatures rather than direct mechanistic biomarkers.

The feature selection process retained features concentrated in the lower portion of the spectrum. For the QRS complex, the selected features were located in the low band (10–50 Hz) with only a single high-band feature (VZ_QRS_High_RelEnergy_mean, 100–150 Hz) and only for the HFrEF vs. HFpEF model. Regarding the ST-T region, the selected features came from both the low (5–15 Hz) and mid (15–50 Hz) bands. The VX_ST_Mid_MeanAmp_std and VX_ST_Low_RelEnergy_mean repolarization features were higher in HF subtypes than in normals and higher in HFrEF than in HFpEF, indicating greater beat-to-beat variability of mid-band amplitude and a larger share of low-band energy concentrated at the lowest frequencies. This X-axis ST pattern reflects differences in left–right ventricular wall repolarization, potentially related to unstable and inconsistent repolarization due to structural substrate changes (such as fibrosis and ischemia). On the contrary, the beat-to-beat variability of the absolute low-band energy on both the X and Y axes (VX_ST_Low_Energy_std and VY_ST_Low_Energy_std) was reduced in HFrEF relative to normals. In the QRS complex, the low-band QRS energy (VX_QRS_Low_Energy_mean) was higher in HFpEF than in HFrEF. As this feature reflects the absolute band energy, it depends on the signal amplitude. Diffuse myocardial fibrosis is known to attenuate ECG voltage independently of left ventricular mass [84]; therefore, the lower low-band energy in HFrEF may indicate voltage reduction rather than redistribution of the spectral content. The relative energy features might be better for assessing the low-frequency shift.

To interpret the predictive ability of the best n^2^ classifier, PFI was utilized. Using the MCC metric for permutation, ’sensitive’ behavior is expected to be observed, as it presents changes across all elements of the confusion matrix. Additionally, due to the small test-set sample, a high variance was anticipated. Indeed, the overall picture shows that the drops in the MCC value differ significantly for each feature. However, the IQRs and mean values show that all the selected features contribute positively to each classification pair. These findings were complimentarily validated by SHAP analysis on sample-level and confirmed the direction and ranking observed with PFI.

For the separation of normals from HF patients, the features of the X-axis repolarization region dominated the importance rankings, in concordance with the described ST-T characteristics and the role of repolarization in HF. The SHAP plots support this pattern, with X-axis features consistently ranking among the top model factors, showing a directional relationship between feature value and impact on model output.

Within the two HF subtypes, VX_QRS_Low_Energy_mean was the most important feature, in line with the expectation that conduction slowing in HFrEF redistributes QRS energy to the lowest band. This was similarly reflected in the SHAP plot, where VX_QRS_Low_Energy_mean ranked at the top, with lower feature values associated with HFrEF pathology, as expected. The single high-band QRS feature is consistent with fibrosis-related microstructural differences between subtypes; however, with only one surviving high-band feature and a comparatively smaller and less consistent SHAP contribution relative to the other features, this remains exploratory.

Across all three pairs of classifications, VX_ST_Mid_MeanAmp_std was assigned high importance, making it a biomarker on which the n^2^ classifier was most consistently based, showing that average instability in high-frequency components during the repolarization phase might have diagnostic value in HF assessment. This was also reflected in the SHAP results, where it was ranked among the highest contributors, strengthening the evidence that it might be a highly influential biomarker. In Appendix E, four representative scenarios are presented for HFpEF and HFrEF patients using SHAP to show the potential feature contributions in different clinical scenarios.

These features presented here are referred to as factors that might be associated with heart failure and the physiology behind the disease rather than as causal factors. In addition, the frequency-band ranges were not selected as the optimal ones to detect HF and should be considered exploratory. Further studies are needed to explore and evaluate different cutoffs for HF assessment.

### 4.3. Comparison with Relevant Published Models

Compared with existing approaches, the normal vs. HFpEF and normal vs. HFrEF models achieved an AUROC of 0.983, which is comparable to or exceeds previously reported results. For HFpEF detection, the AUROC range was 0.79–0.89 [12,13,14,15], and for HFrEF detection, the AUROC was 0.844–0.93 [16,17]. A direct comparison is limited by differences in population size, signal modalities and HF definition criteria. Different origins of healthy and HF patients and a lack of external validation should also be considered in this section.

Regarding the discrimination between HFrEF and HFpEF, the present model did not reach the performance reported by [21], although this comparison should be interpreted with caution. The two studies used an identical source and relied on feature-based machine learning, but they differ in scope. Also, ref. [21] did not include healthy subjects and stratified patients regardless of rhythm, in contrast with the current work, which was restricted to individuals in sinus rhythm. Additionally, in [21], insightful findings about feature importance are not reported, hindering the interpretation of the results in general and the key differences during the classification task between the two studies. Notably, ref. [21] was not externally validated—similarly to this study—and evaluation on independent datasets is required to establish generalizability. In a different cohort, ref. [19] also addressed a three-classification problem within a population selected for prior myocardial infarction or ischemic heart disease, without a healthy control group, and used long-duration Holter recordings, while the present work addressed a binary distinction within a more heterogeneous HF cohort that also included healthy controls. However, convergence was observed at the feature level in the present study. Ref. [19] identified QTc, QRS, ST-T and TP intervals as the most important features, relating this to ventricular remodeling and repolarization abnormalities. The PFI analysis in the present study similarly identified the ST-T- and QRS-region spectral features as important markers of HF. An interesting finding is that both [19,20] found that the HF subtype classification accuracy varies substantially by time of day. This has direct implications for the design of outpatient monitoring protocols and may explain some of the heterogeneity in the results between retrospective studies that do not take into account the time at which the ECG was recorded.

It is also worth mentioning related work regarding wavelet-based ECG feature approaches for LV dysfunction, as mentioned above. In [23], wavelet ECG features and clinical variables were combined to estimate myocardial relaxation velocity and discriminate systolic and diastolic LV dysfunction in a multicenter cohort of 1202 subjects, achieving AUROCs of 0.75–0.94 across internal and external test sets. Similarly, ref. [25] combined wavelet energy-based features (ewECG) with the ARIC HF clinical score to detect subclinical systolic and diastolic LV dysfunction in 398 individuals at risk of HF, achieving an AUROC of 0.83 in an independent test set of 111 participants. An earlier study from the same group utilized ewECG features and a RF classifier for the screening of 319 asymptomatic subjects. External validation showed a sensitivity of 93% and an AUROC of 0.76, with the authors estimating that this could reduce unnecessary echocardiography by 38%. In [26], similar ewECG features were used in combination with clinical variables and NT-proBNP to detect subclinical LV dysfunction in patients with type 2 diabetes mellitus, achieving an AUROC of 0.81, significantly outperforming NT-proBNP and the ARIC HF score alone, with AUROC values of 0.56 and 0.67, respectively, in a validation cohort of 97 participants. These studies support the notion that traditional machine learning models based on wavelet-based ECG features can achieve performance comparable to that of more complex deep learning approaches while offering greater interpretability, consistent with the feature-based approach adopted in this work. However, these studies incorporated clinical data alongside ECG features, which the present study did not include, and they targeted subclinical LV dysfunction rather than the HF subtype distinction addressed here.

An important point is that the EF threshold definitions vary across studies, which limits direct comparability. In [19], the ASE/EACVI criteria were adopted (HFrEF < 50%; HFmrEF, 50–55%; HFpEF > 55%), differing from the ESC/ACC-AHA thresholds used in this work. In addition, ref. [85] showed that the AUROC for low-EF classification varied significantly with the chosen threshold (35–50%). This suggests that the performance metrics for EF classification tasks are threshold-dependent to some extent, and comparisons between studies using different EF thresholds should be evaluated circumspectly.

### 4.4. Dataset Heterogeneity

Normals and HF patients come from different databases, and differences in hardware, conditions and acquisition protocol might introduce bias to a model. First, in Table 1, it is presented that normal and HF patients have an age gap, which is backed up by the HF epidemiology. In [4], it was reported that HF affects approximately 1% of those under 55 years and over 10% of those aged 70 or older. This gap is difficult to avoid when relying only on publicly available databases with different origins.

Sex differences were not found to be significant, as normal and HF samples had nearly the same sex percentages, and HFpEF had a 10% greater female population than the other two classes. This difference is attributed to the difference in HF subtype epidemiology; HFpEF is more common in females, and HFrEF is more common in males [4].

The drop in the HFrEF vs. HFpEF task might raise some flags, but HF is a complex syndrome characterized by comorbidity; thus, distinguishing HF subtypes from each other is more complicated than normals vs. HF. Also, the small number of dataset in this study is not ideal for fully investigating this kind of heterogeneity of HF.

Additionally, the most significant features selected for each task and the feature importance analysis are reasonable and in concordance with literature findings regarding the underlying pathophysiological mechanisms and what could really differ among the three classes. In addition, the findings of the Appendix A.3 suggest that model predictions were mostly driven by physiological factors rather than dataset-specific differences; however, with such a small number of HF patients from PTB, further evaluation on larger cohorts is essential to ensure robustness and reliability.

### 4.5. Power Analysis Results

Post hoc power analysis showed that all three binary classifiers, achieved 0.84 power to detect AUROC ≥ 0.65 and ≥0.98 power at AUROC ≥ 0.70. The combined n^2^ classifier had ≥0.99 power to confirm accuracy ≥ 0.65 against the NIR (0.531). These results, together with the 95% CIs from 100 repeated nested cross-validation iterations (Table 6 and Table 7), the metrics from the screening task (Table 8) and the EPV ratios confirming model stability (Appendix D) suggest that the sample size (and the HFpEF group) is sufficient for the reported analyses.

### 4.6. Limitations and Future Steps

Although the results suggest that this method can contribute to HF assessment, several limitations should be considered. First, the dataset was relatively small and imbalanced, particularly in the HFpEF group, increasing the risk of overfitting. Although SMOTE was deployed and assisted in the classification process, imbalance can still reduce the sensitivity of the minority class (HFpEF). Second, healthy controls and HF patients were derived from different open-access databases, introducing potential domain-shift bias related to the acquisition protocols, recording conditions, and population characteristics. Third, the study included only individuals with sinus rhythm in order to isolate morphological changes from rhythmic abnormalities. Thus, the selected features and models have not been validated for patients with arrhythmias, frequent ectopy or paced rhythm—which are common in real-world HF populations; applying them in practice would likely require a prior rhythm inspection step. Also, beyond subtype classification, a recent study using paired recordings showed that HF status changes over time [86], suggesting that long-term assessment might provide diagnostic information and should not be limited to a single time point. Finally, this study uses cross-validation and tests with multiple splits, but the models were not validated in an external data source.

Future studies should include external validation of the models to assess their performance and robustness with current findings. Additionally, the development of new models with 12-lead ECGs is a reasonable extension, as this lead system is intrinsically linked to the actual clinical setting. Demographics and clinical characteristics of a patient, such as systolic/diastolic pressure, blood glucose levels, and natriuretic peptides, constitute a notable addition in the detection of HF [87] and linking them with ECG markers.

## 5. Conclusions

This study presented a machine learning approach for HF assessment in subjects with SR and highlights the significance of ECG in clinical practice. The main observations suggest that CWT-derived ECG/VCG features may contain relevant information for HF assessment in patients with SR. The strongest findings support the potential role of ECG/VCG-based machine learning as an accessible screening tool for identifying individuals who may require further HF evaluation. The interesting finding of a common feature among the three classifiers (VX_ST_Mid_MeanAmp_std) should be explored in future studies. However, due to the small sample size, class imbalance, use of separate databases, and the preliminary nature of the findings (lack of external validation), the proposed approach should not yet be considered a standalone diagnostic tool.

Through effective diagnosis and screening of patients with HF, the field of personalized medicine will gain fertile ground for further advancements. Accurate identification of the phenotypic groups for each subtype will significantly assist in creating solutions adapted to the needs of each patient, thereby improving their quality of life and life expectancy. 

## Figures and Tables

**Table 1:** Demographics per class.

Class	Age	Male	Female	Available (*n*)
normal	40.19 ± 14.08	60 (75.0%)	20 (25.0%)	73/80
HFpEF	59.78 ± 12.69	32 (64.0%)	18 (36.0%)	50/50
HFrEF	60.33 ± 11.70	112 (76.0%)	35 (23.8%)	147/147

**Table 2:** HRV features calculated from VX lead and their description.

HRV Feature	Description
Mean RR (ms)	Average time interval between consecutive R-peaks
SDNN (ms)	Standard deviation of all normal-to-normal (NN) intervals, capturing sympathetic and parasympathetic influences
RMSSD (ms)	Root mean square of successive differences between adjacent NN intervals
NN50	Number of pairs of successive NN intervals that differ by more than 50 ms
PNN50 (%)	Percentage of NN50 relative to the total number of NN intervals
Mean HR (bpm)	Average heart rate over the recording period
STD HR (bpm)	Standard deviation of heart-rate values
Min HR (bpm)	Lowest heart rate recorded during the recording period
Max HR (bpm)	Highest heart rate recorded during the recording period
SD1	Standard deviation of the short-term (beat-to-beat) variability—related to RMSSD
SD2	Standard deviation of long-term variability of NN intervals
SD1/SD2	Ratio of short-term to long-term variability
Sample entropy	Measure of irregularity and complexity of the RR interval time series

**Table 3:** Frequency bands for the QRS complex and ST-T region.

QRS Bands	ST-T Bands
10–50 Hz (Low)	5–15 Hz (Low)
50–100 Hz (Mid)	15–50 Hz (Mid)
100–150 Hz (High)	
150–200 Hz (VeryHigh)	

**Table 4:** CWT features calculated from the 3 leads from each band.

CWT Feature	Description
Energy	Measures the signal power in each wavelet band
Relative Energy	Measures the band relative energy to total energy across bands
Entropy	Measures the complexity/irregularity of the wavelet coefficients in the band
Mean Amplitude	Measures the mean absolute amplitude of the wavelet coefficients in the band
Maximum Amplitude	Measures the maximum absolute amplitude of the wavelet coefficients in the band

**Table 5:** Feature selection results. Features in bold are the common ones selected between the models.

Normal vs. HFpEF	Normal vs. HFrEF	HFrEF vs. HFpEF
**VX_ST_Low_RelEnergy_mean**	**VX_ST_Low_RelEnergy_mean**	VZ_QRS_High_RelEnergy_mean
**VX_QRS_Low_RelEnergy_mean**	**VX_QRS_Low_RelEnergy_mean**	VX_QRS_Low_Energy_mean
**VX_ST_Mid_MeanAmp_std**	**VX_ST_Mid_MeanAmp_std**	**VX_ST_Mid_MeanAmp_std**
	VY_ST_Low_Energy_std	
	VZ_QRS_Low_Energy_mean	
	VX_ST_Low_Energy_std	

**Table 6:** Performance metrics for normal vs. HFpEF model for 100 classification repetitions.

Metric	Mean	95% CI
Accuracy	0.923	0.915–0.932
Precision	0.926	0.911–0.942
Recall	0.880	0.861–0.899
Specificity	0.951	0.939–0.962
F1 score	0.897	0.885–0.909
AUROC	0.983	0.979–0.987
Balanced accuracy	0.915	0.905–0.925
MCC	0.843	0.825–0.861

**Table 7:** Performance metrics for normal vs. HFpEF and HFrEF vs. HFpEF models for 100 classification repetitions.

Model	Metric	Mean	95% CI
Normal vs. HFrEF	Accuracy	0.937	0.930–0.943
	Precision	0.971	0.964–0.977
	Recall	0.932	0.924–0.940
	Specificity	0.945	0.932–0.958
	F1 score	0.950	0.945–0.955
	AUROC	0.983	0.980–0.986
	Balanced accuracy	0.939	0.932–0.945
	MCC	0.867	0.854–0.879
HFrEF vs. HFpEF	Accuracy	0.669	0.654–0.684
	Precision	0.418	0.402–0.434
	Recall	0.749	0.724–0.774
	Specificity	0.643	0.625–0.661
	F1 score	0.533	0.516–0.550
	AUROC	0.766	0.749–0.782
	Balanced accuracy	0.696	0.680–0.711
	MCC	0.345	0.318–0.373

**Table 8:** Performance metrics from patient screening for 3 test sets.

Class	Precision	Recall	F1 Score	AUROC
normal	0.84 ± 0.046	0.88 ± 0.000	0.86 ± 0.024	0.99 ± 0.003
HFrEF	0.85 ± 0.056	0.76 ± 0.031	0.80 ± 0.042	0.87 ± 0.037
HFpEF	0.47 ± 0.100	0.60 ± 0.163	0.53 ± 0.125	0.79 ± 0.083
Macro Avg.	0.72 ± 0.067	0.74 ± 0.065	0.73 ± 0.063	

**Figure A4:**
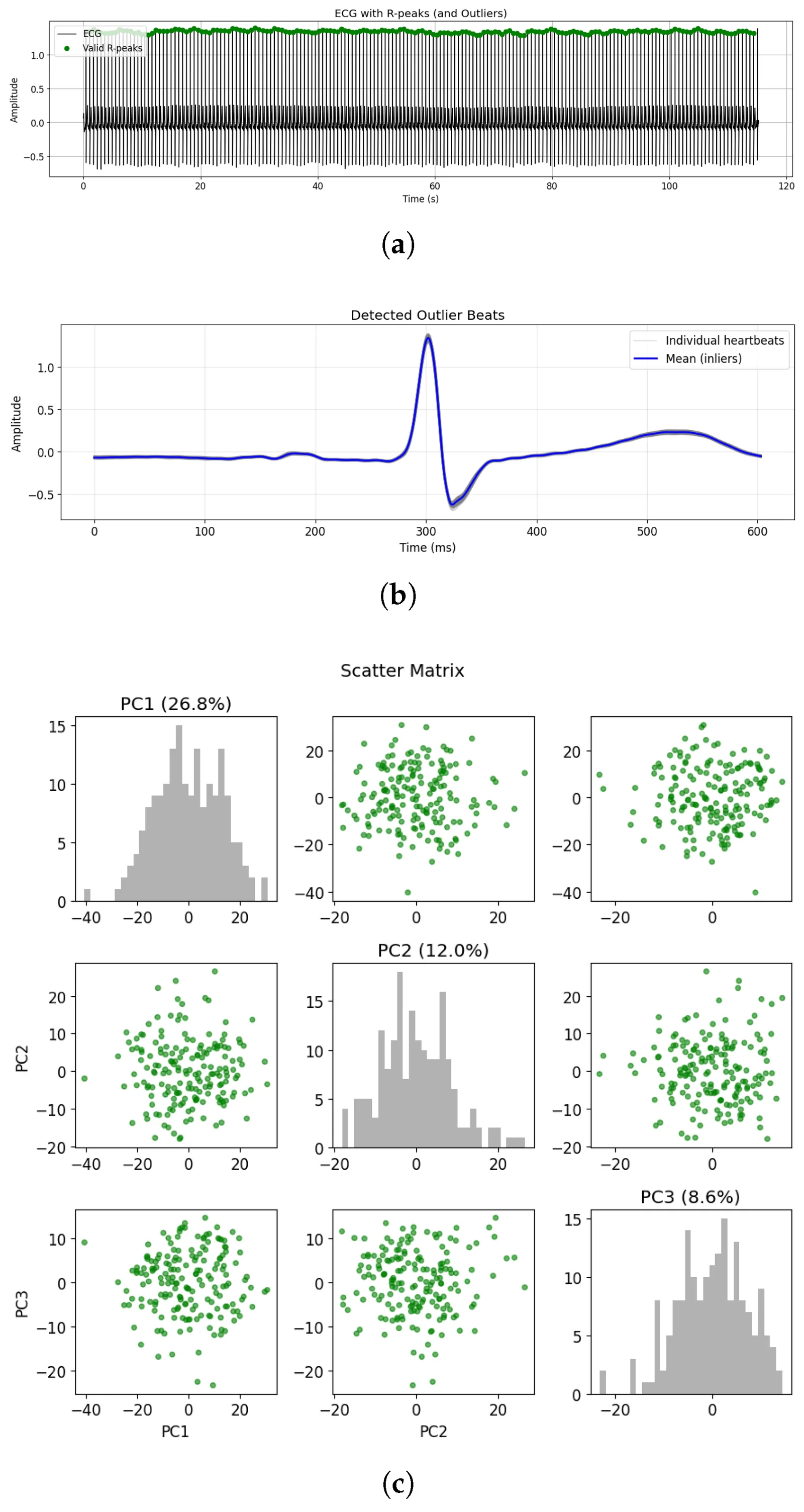
Outlier detection evaluation on a clean signal. (**a**) Visualization of R peaks (outliers not found) across the whole signal; (**b**) All extracted heartbeats plotted together—each gray line represents each heartbeat and the blue line the average heartbeat; (**c**) Scatter matrix of PC1, PC2 and PC3. Diagonal panels show the distribution (histogram) of each PC and off-diagonal panels show pairwise scatter plots (outliers were not found).

**Figure A6:**
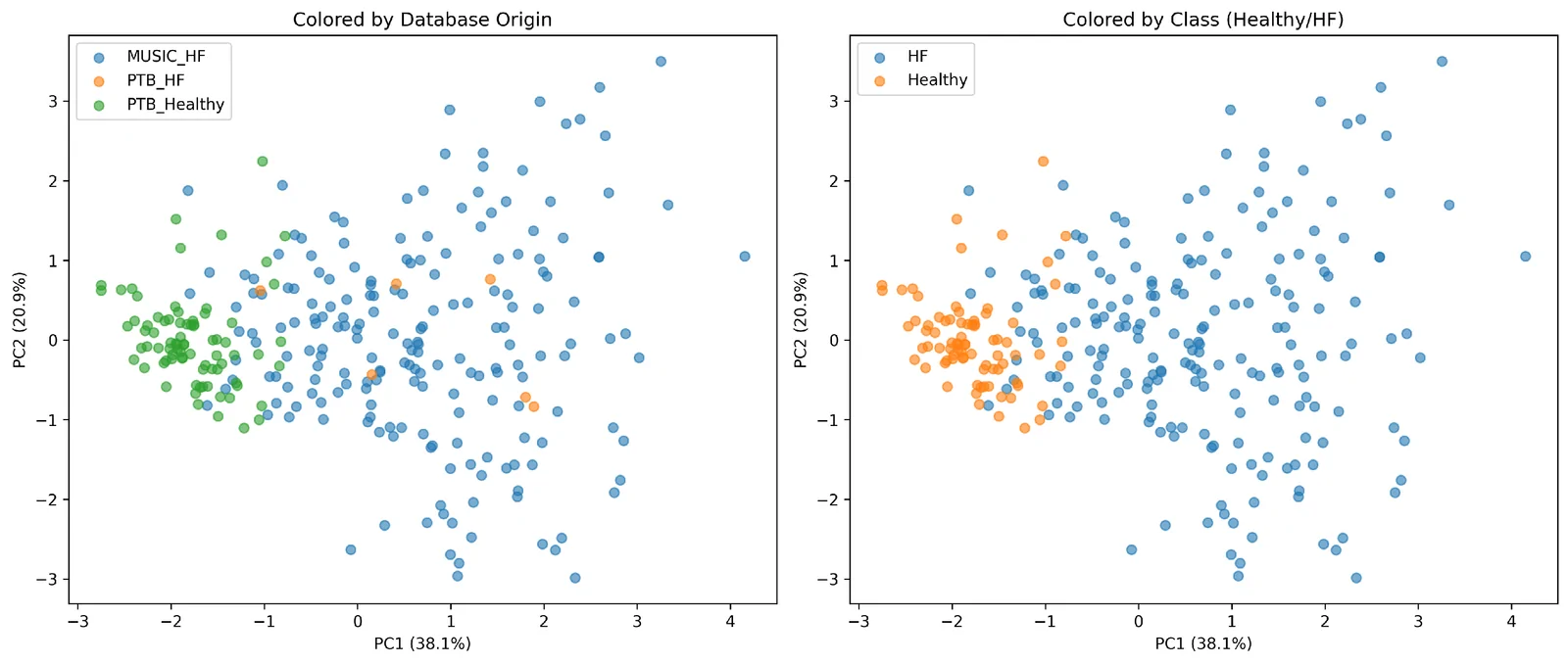
Batch effect analysis including healthy subjects from PTB and HF patients from MUSIC and PTB—PC1 + PC2.

**Figure A7:**
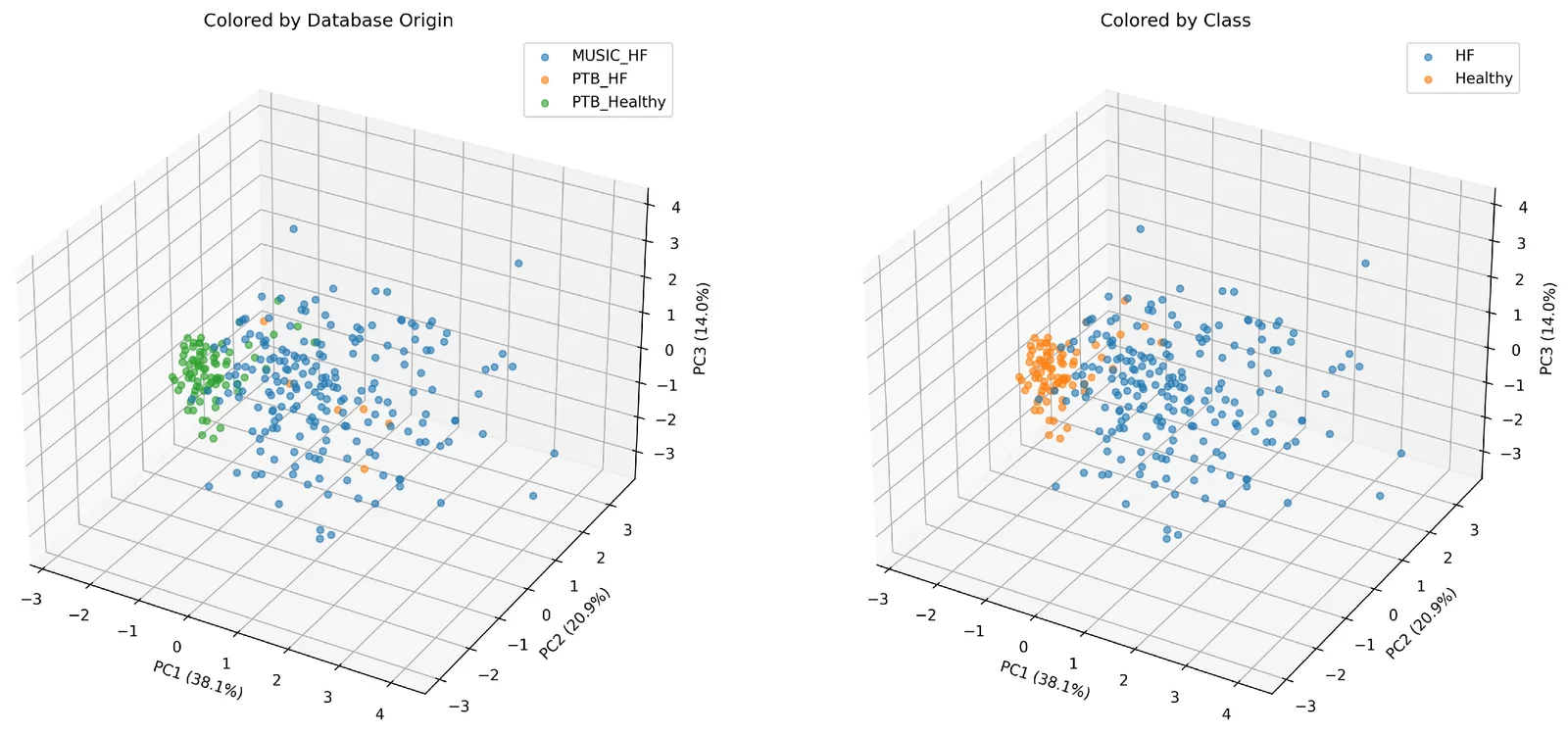
Batch effect analysis including healthy subjects from PTB and HF patients from MUSIC and PTB—PC1 + PC2 + PC3.

**Figure A8:**
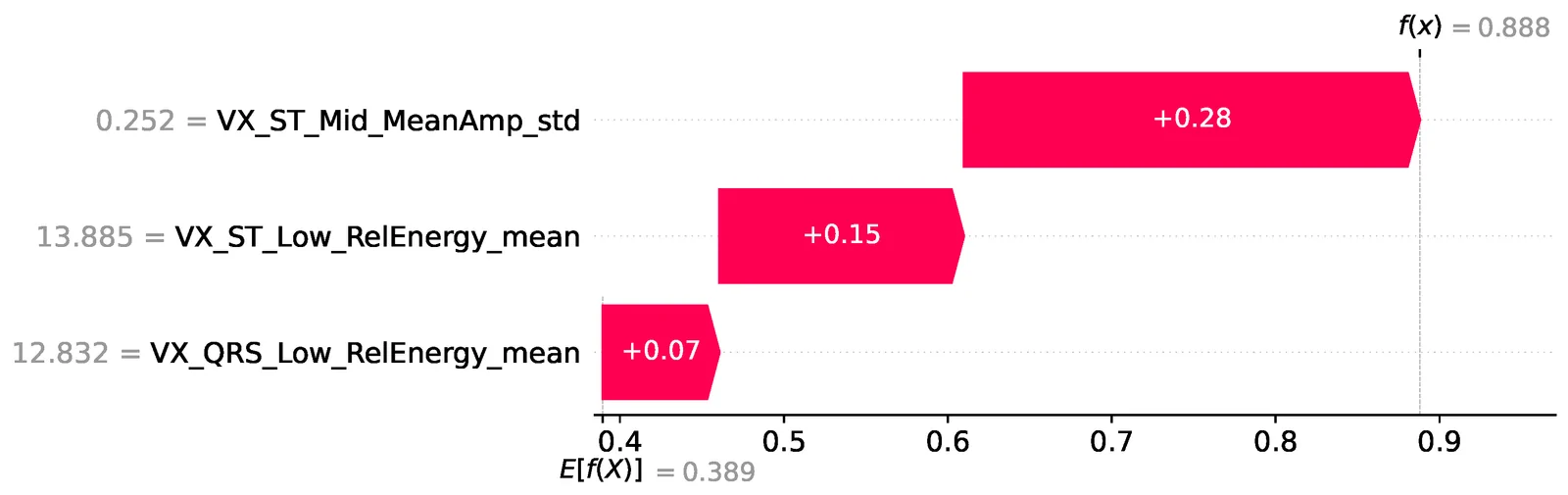
Waterfall SHAP plot—HFpEF patient 1. Red color indicates features that push the prediction toward the positive class (HFpEF).

**Figure A9:**
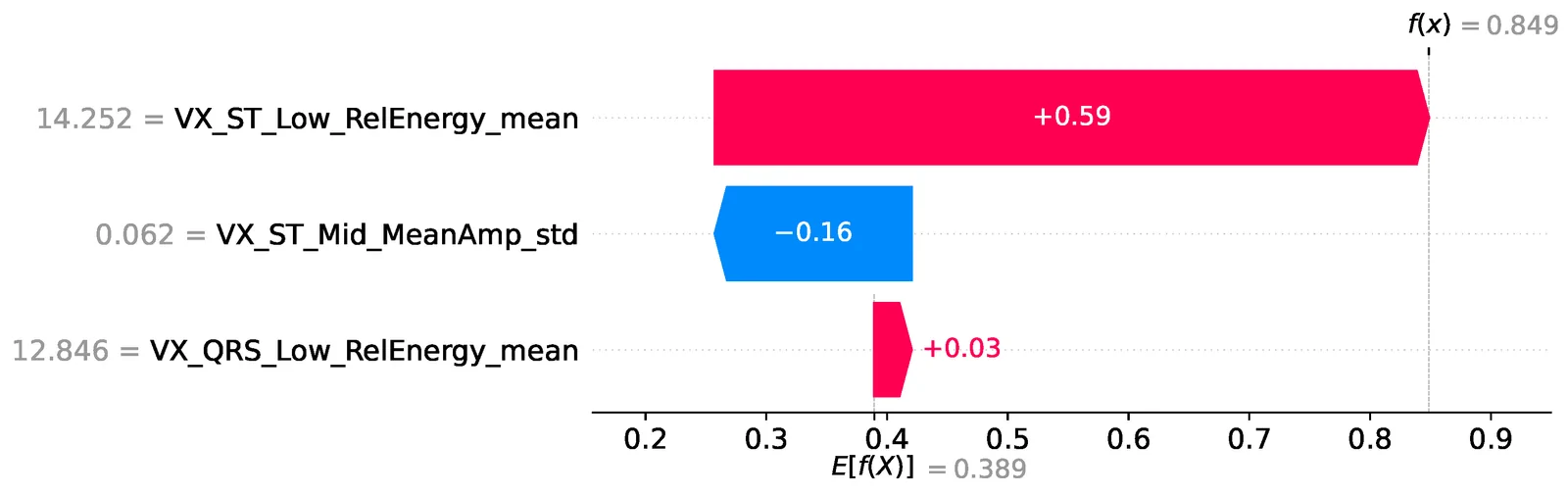
Waterfall SHAP plot—HFpEF patient 2. Red color indicates features that push the prediction toward the positive class (HFpEF) while blue color indicates features that push the prediction toward the negative class (normal).

**Figure A10:**
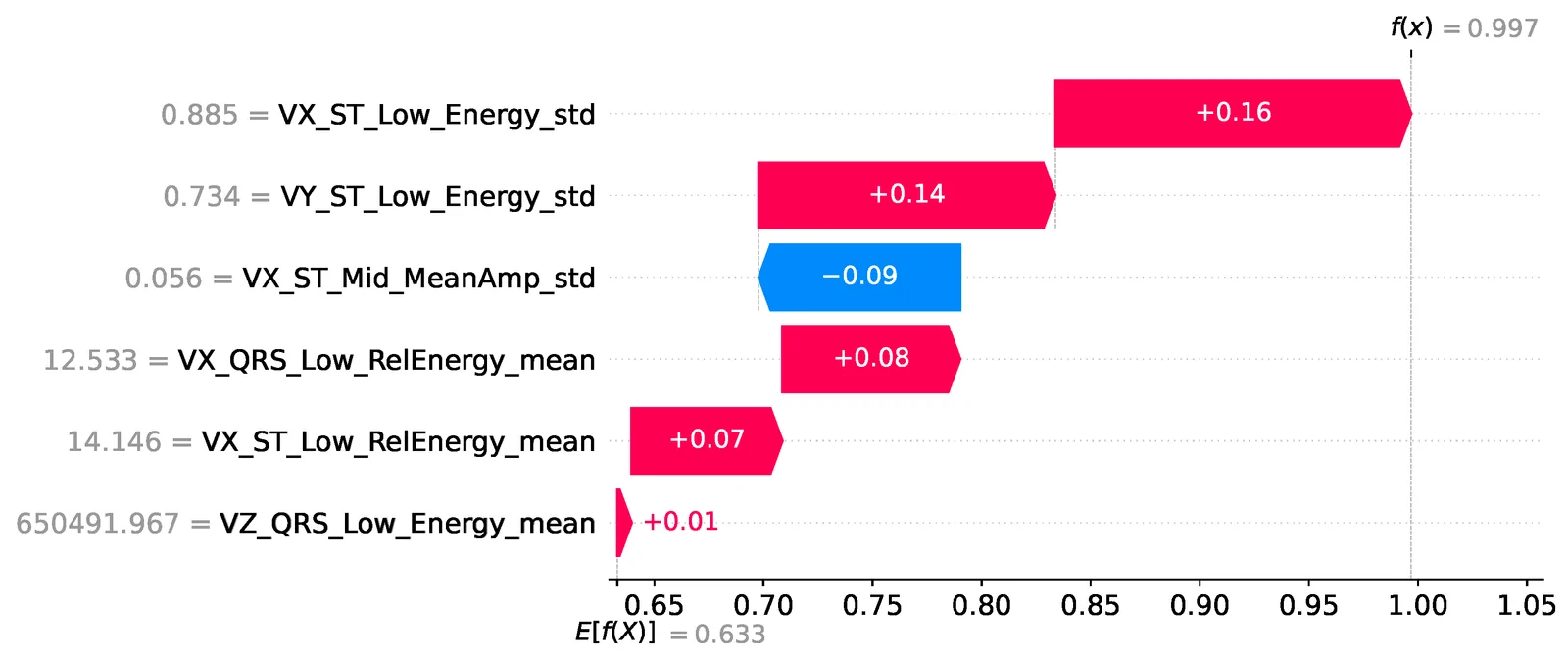
Waterfall SHAP plot—HFrEF patient 1. Red color indicates features that push the prediction toward the positive class (HFrEF) while blue color indicates features that push the prediction toward the negative class (normal).

**Figure A11:**
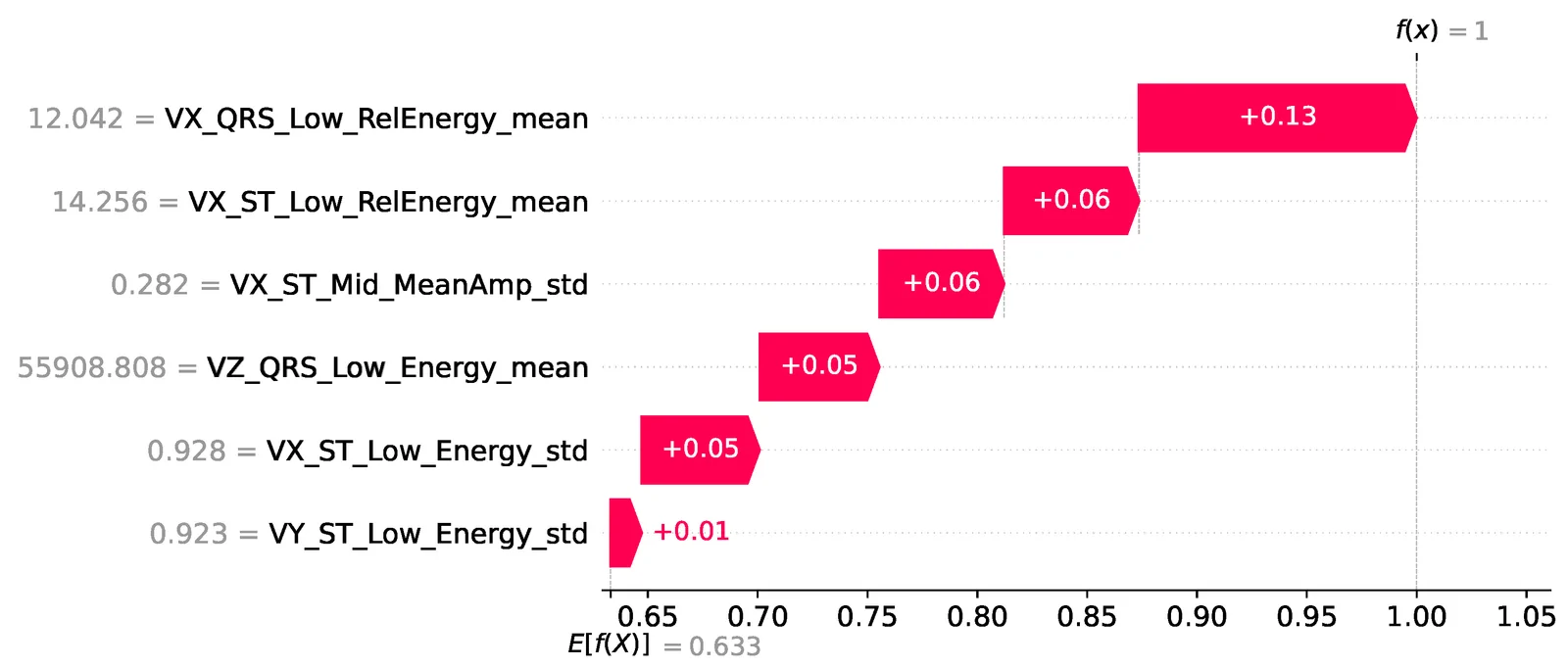
Waterfall SHAP plot—HFrEF patient 2. Red color indicates features that push the prediction toward the positive class (HFrEF) while blue color indicates features that push the prediction toward the negative class (normal).

## Data Availability

This study used exclusively publicly available data from PhysioNet—specifically, the PTB Diagnostic ECG Database (https://www.physionet.org/content/ptbdb/1.0.0/, accessed on June 2026) and MUSIC (Sudden Cardiac Death in Chronic Heart Failure) (https://www.physionet.org/content/music-sudden-cardiac-death/1.0.1/, accessed on June 2026) under Open Database License (ODbL) v1.0.

## Supplementary Materials

The following supporting information can be downloaded at: https://www.mdpi.com/article/10.3390/jpm16090480/s1, Data S1: Performance metrics for the remaining models of normal vs. HFpEF classification task. Data S2: Performance metrics for the remaining models of normal vs. HFrEF classification task. Data S3: Performance metrics for the remaining models of HFrEF vs. HFpEF classification task. Data S4: Performance metrics for every iteration of normal vs. HFpEF classification task. Data S5: Performance metrics for every iteration of normal vs. HFrEF classification task. Data S6: Performance metrics for every iteration of HFrEF vs. HFpEF classification task. Data S7: Statistical significance of HRV features. Data S8: AUC values across 30 training iterations using Logistic Regression model.

## Abbreviations

The following abbreviations are used in this manuscript:

HF	Heart Failure
EF	Ejection Fraction
HFpEF	Heart Failure with preserved Ejection Fraction
HFrEF	Heart Failure with reduced Ejection Fraction
HFmrEF	Heart Failure with mildly reduced Ejection Fraction
LVEF	Left Ventricular Ejection Fraction
BNP	B-type Natriuretic Peptide
NT-proBNP	N-Terminal pro-B-type Natriuretic Peptide
LV	Left Ventricular
SR	Sinus Rhythm
VT	Ventricular Tachycardia
AF	Atrial Fibrillation
PVC	Premature Ventricular Contraction
CHF	Chronic Heart Failure
LBBB	Left Bundle Branch Block
LVH	Left Ventricular Hypertrophy
AI-DSS	Artificial Intelligence Decision Support System
ECG	Electrocardiogram
VCG	Vectorcardiogram
HRV	Heart Rate Variability
CWT	Continuous Wavelet Transform
DWT	Discrete Wavelet Transform
HR	Heart Rate
NN	Normal-to-Normal (interval)
MAD	Median Absolute Deviation
SVM	Support Vector Machine
RF	Random Forest
XGBoost	eXtreme Gradient Boosting
PCA	Principal Component Analysis
kNN	k-Nearest Neighbors
DBSCAN	Density-Based Spatial Clustering of Applications with Noise
SMOTE	Synthetic Minority Over-sampling Technique
RENT	Repeated Elastic Net Technique
MCC	Matthews Correlation Coefficient
AUROC	Area Under the Receiver Operating Characteristic curve
CM	Confusion Matrix
PFI	Permutation Feature Importance
CI	Confidence Interval

## Appendix A

### Appendix A.1. Denoising Pipeline

The effect of each denoising pipeline step—for a PTB and a MUSIC subject—is presented in Figure A1.

For the same signal from PTB white noise (SNR = 15 dB), power-line interference at 50, 100 and 150 Hz; baseline wander; and high-amplitude spikes were added artificially. The correlation coefficient was calculated before and after denoising and resulted in ρ=0.5518 and ρ=0.9818, respectively. This is also depicted in Figure A2 and Figure A3.

### Appendix A.2. Outlier Detection

The performance of outlier detection is presented in Appendix A.2 for a clean signal and in Figure A5 for a contaminated signal.

### Appendix A.3. Batch Effect Calculation

To calculate if there is batch effect between the two databases, six HF patients were included from the PTB database—the only HF patients in the database. These patients were not included in this study’s approach because their EF information was absent. Using the selected features from RENT that correspond to normal vs HF classification tasks (six in total), various tests were conducted to ensure the classification was driven by physiology rather than dataset-specific factors.

First, using PCA to visualize samples in 2D and 3- space, it was shown that there is no visual boundary between MUSIC HF and PTB HF patients, while normals (healthy patients) form their own cluster (Figure A6 and Figure A7).

Second, to quantify the separation of classes beyond PCA, the Silhouette score [88] and PERMANOVA test [89] were applied; the silhouette score was used as a descriptive measure to evaluate the similarity of each sample relative to the other samples within its own group [88], and PERMANOVA was used as a non-parametric statistical test computing a pseudo-F statistic that determines the statistical significance by randomly permuting group labels [89]. This approach was applied on (**a**) HF patients from PTB and MUSIC to isolate the database effect, and (**b**) healthy individuals from PTB and HF patients from PTB to isolate the class effect. For the PERMANOVA test, the scikit-bio library was used [90]. The results presented in Table A1 show that HF samples from the two databases do not show separation in silhouette score and non-statistically significant separation in PERMANOVA test. On the other hand, healthy and HF patients from PTB show a moderate silhouette score and a highly statistically significant PERMANOVA result.

**Table A1:** Silhouette score and PERMANOVA results.

Comparison	# Group A	# Group B	Silhouette Score	PERMANOVA (Pseudo-F)	PERMANOVA (*p*-Value)	Permutations
PTB HF vs. MUSIC HF	6	197	−0.073	0.114	0.996	999
PTB healthy vs. PTB HF	80	6	0.535	24.572	0.001	999

Note: # = number of samples.

Third, a logistic regression model was trained on the selected features to predict the origin of the database. For this training scheme, leave-one-out cross-validation (LOOCV) was utilized following a procedure of 30 iterations, each using the 6 HF patients from PTB and a randomly selected subset of 6–10 samples from (a) MUSIC HF patients and (b) PTB healthy patients (same rationale as above), using AUC as the performance metric (mean value, median and std). Results in Table A2 present very low mean AUROC values when isolating the database effect and very high values when isolating the class effect, meaning that the database origin of samples in HF patients cannot be distinguished and physiology-related differences are highly detected in samples from the same database. Results for each iteration can be found in Data S8 (see Supplementary Materials).

**Table A2:** Logistic regression model performance.

Comparison	AUROC (Mean)	AUROC (Median)	AUROC (Std)
PTB HF vs. MUSIC HF	0.268	0.266	0.147
PTB healthy vs. PTB HF	0.974	0.983	0.038

Additionally, the performance of the trained models—(a) normal vs. HFpEF and (b) normal vs. HFrEF—was assessed by inferring the class of the six HF patients from PTB. Results in Table A3 and Table A4 show that none of the six patients was predicted as normal, a finding that is consistent with the previous results of this section.

**Table A3:** Inference using normal vs. HFpEF model on PTB HF patients.

Model	Sample	Probability
Normal vs. HFpEF	Patient 1	0.964
	Patient 2	0.997
	Patient 3	0.899
	Patient 4	0.992
	Patient 5	1.000
	Patient 6	0.997

**Table A4:** Inference using normal vs. HFrEF model on PTB HF patients.

Model	Sample	Probability
Normal vs. HFrEF	Patient 1	0.733
	Patient 2	0.997
	Patient 3	0.992
	Patient 4	0.954
	Patient 5	1.000
	Patient 6	0.990

## Appendix B

### Hyperparameter Tuning

**Table A5:** Hyperparameter ranges explored in GridSearchCV.

Model	Hyperparameter	Values
SVM	C	0.01, 0.1, 1, 10, 100
	kernel	linear, rbf
	gamma	0.001, 0.01, 0.1, 1
	class_weight	None, balanced
RF	n_estimators	100, 300
	max_depth	None, 5, 10, 20
	min_sample_split	2, 5
	class_weight	None, balanced
XGB	n_estimators	50, 100, 200
	learning_rate	0.1, 0.01
	max_depth	3, 6
	scale_pos_weight	1, 2.94

**Table A6:** The selected best models for each classification task with their hyperparameters.

Classfication Task	Model	SMOTE	Hyperparameters
Normal vs. HFpEF	SVM	Yes	C = 0.1
			kernel = rbf
			gamma = 0.1
Normal vs. HFrEF	SVM	Yes	C = 1
			kernel = rbf
			gamma = 0.1
HFrEF vs. HFpEF	SVM	Yes	C = 10
			kernel = rbf
			gamma = 0.1

## Appendix C

### Appendix C.1. Confidence Intervals for Scalar Performance Metrics

For each scalar performance metric, the mean and 95% CI were computed as follows:

(A1) $$CI_{95\%}=\bar{x}\pm 1.96\cdot \frac{s}{\sqrt{n}}$$

where $\bar{x}$ is the mean, *s* is the standard deviation using Bessel’s correction, n=100 is the number of iterations and 1.96 is the critical value of the standard normal distribution for a 95% CI. For n=100, we can assume normality due to the Central Limit Theorem.

### Appendix C.2. Confidence Intervals for AUROC

Each iteration’s curve was interpolated onto a common grid of 100 equally spaced false-positive-rate (FPR) values, and the mean true positive rate (TPR) with its standard error was computed at each grid point—a method known as vertical averaging [91,92]. Pointwise 95% CIs were constructed using 1.96×SE clipped to [0, 1].

### Appendix C.3. Confusion Matrix Summary

The aggregated CMs were obtained by summing recall and specificity as described above, and false-negative and false-positive rates were calculated as 1−recall and 1−specificity with the corresponding CIs ($lower^{\prime }=1-upper$ and $upper^{\prime }=1-lower$).

## Appendix D

### Appendix D.1. Framework

The study cohort was imbalanced across the three classes, and classification was performed using three binary classifiers and combined n^2^ classifiers. The self-sufficiency of the sample size was evaluated using multiple approaches: (a) theoretical power for each binary classifier’s ability to discriminate, (b) the theoretical power of the n^2^ classifier’s accuracy, (c) the theoretical precision of class-specific performance estimates and (d) a model-stability check—EPV—for the HFpEF group (the class with the fewest samples).

### Appendix D.2. Binary Classifier Power

Statistical power for each binary classifier was assessed using the Hanley and McNeil method [72], which relates the AUROC to its sampling variance under the null hypothesis and under a specified AUROC (*A*). For classes of size $n_{1}$ and $n_{2}$,

(A2) $$\begin{array}{cc} V_{0} & =\frac{n_{1}+n_{2}+1}{12n_{1}n_{2}} \end{array}$$

(A3) $$\begin{array}{cc} V_{A} & =\frac{A\left(1-A\right)+\left(n_{1}-1\right)\left(Q_{1}-A^{2}\right)+\left(n_{2}-1\right)\left(Q_{2}-A^{2}\right)}{n_{1}n_{2}}, \end{array}$$

with $Q_{1}=A/\left(2-A\right)$ and $Q_{2}=2A^{2}/\left(1+A\right)$. The achieved power for a two-sided test at α=0.05 was computed as

(A4) $$\Phi [\frac{\left(A-0.5\right)-z_{a/2}\sqrt{V_{0}}}{\sqrt{V_{A}}}]$$

accoridng to Obuchowski and McClish [73]. Table A7 shows that the weakest pair, normal vs. HFpEF, still reaches approximately 84% power at AUROC = 0.65 and 98% at AUROC = 0.70.

**Table A7:** Binary classification power to detect AUROC > 0.5.

Pair	$n_{1}$	$n_{2}$	AUROC 0.60	AUROC 0.65	AUROC 0.70	AUROC 0.75	AUROC 0.80
Normal vs. HFpEF	80	50	0.481	0.839	0.999	1.000	1.000
Normal vs. HFrEF	80	147	0.702	0.966	0.984	1.000	1.000
HFrEF vs. HFpEF	147	50	0.563	0.911	0.997	1.000	1.000

To achieve the minimum required power of 80%, all observed pair sizesmeet at AUROC ≥ 0.70, as shown in Table A8.

**Table A8:** Minimum sample size for 80% power (same class ratio per pair).

Pair	Target AUROC	Min. $n_{1}$	Min. $n_{2}$	Min. Total
Normal vs. HFpEF	0.65	74	46	120
Normal vs. HFpEF	0.70	40	25	65
Normal vs. HFrEF	0.65	45	82	127
Normal vs. HFrEF	0.70	25	46	71
HFrEF vs. HFpEF	0.65	109	37	146
HFrEF vs. HFpEF	0.70	62	21	83

### Appendix D.3. n^2^ Classifier Power

For the combined decision, power was assessed as a one-sample test of overall accuracy against NIR = 147/277 = 0.531, using a binomial test at α=0.05, cross-checked against the approximation:

(A5) $$\Phi [\frac{|p_{1}-p_{0}|-z_{a}\sqrt{p_{0}\left(1-p_{0}\right)/N}}{\sqrt{p_{1}\left(1-p_{1}\right)/N}}]$$

**Table A9:** Power of overall accuracy against NIR.

Target Accuracy	Exact Power	Normal Approximation Power
0.55	0.134	0.158
0.60	0.719	0.752
0.65	0.990	0.993
0.70	1.000	1.000
0.75	1.000	1.000
0.80	1.000	1.000

**Table A10:** Minimum N for 80% power.

Target Accuracy	Minimum M	Sufficiency of Current N = 277
0.60	320	No (72% power)
0.65	109	Yes (100% power)
0.70	52	Yes (100% power)
0.75	30	Yes (100% power)
0.80	20	Yes (100% power)

### Appendix D.4. Theoretical Precision of Class-Specific Estimates

Theoretical precision was assessed through 95% Wilson-score confidence intervals for the sensitivity metric, assuming a true rate of 0.80.

**Table A11:** Theoretical 95% precision.

Class	n	Theoretical 95% CI (assumed *p* = 0.80)	Half-Width
Normal	80	[0.700, 0.873]	±0.087
HFrEF	147	[0.728, 0.857]	±0.064
HFpEF	50	[0.670, 0.888]	±0.109

### Appendix D.5. Model Stability—EPV

Overfitting risk was assessed due to the small number of HFpEF samples. EPV was calculated for each binary classifier as minority-class size divided by the number of estimated model parameters, following the conventional EPV ≥ 10 rule [74]. Table A12 shows that all three models exceed the conventional threshold, indicating that the number of the selected features for each task is adequate.

**Table A12:** EPV by binary classification task.

Pair	Minority Class (n)	Features (*p*)	Parameters (*p* + 1)	EPV
Normal vs. HFpEF	HFpEF (50)	3	4	12.5
Normal vs. HFrEF	normal (80)	6	7	11.4
HFrEF vs. HFpEF	HFpEF (50)	3	4	12.5

## Appendix E

### Potential Clinical Scenarios and Feature Contribution

To present potential clinical scenarios of HFpEF and HFrEF, individual scenarios illustrate how the the selected features might contribute in decision making in clinical settings.

In Figure A8 and Figure A9, both patients have HFpEF and were correctly diagnosed by the model during internal validation. In patient 1 (Figure A8), all three features contribute to the positive direction, with VX_ST_Mid_MeanAmp_std as the dominant factor. This might suggest broadly consistent abnormalities across both depolarization and repolarization regions. In patient 2 (Figure A9), the prediction is driven almost entirely by VX_ST_Low_RelEnergy_mean, while VX_ST_Mid_MeanAmp_std pushes the model in the opposite direction, which might suggest that abnormalities are more localized to the low-frequency repolarization region.

In Figure A10 and Figure A11, both patients have HFrEF and were correctly diagnosed by the modelduring internal validation. In patient 1 (Figure A10), five of the six features push the prediction towards HFrEF (mainly VX_ST_Low_Energy_std and VY_ST_Low_Energy_std), while VX_ST_Mid_MeanAmp_std pulls in the opposite direction. While this opposition is relatively low (0.056), this might suggest that for these patients, low-frequency components in the repolarization region might carry more diagnostic information than high-frequency components. In patient 2 (Figure A11), the direction of all features align, which might suggest conductance abnormalities both in depolarization and repolarization regions.

In all four cases, predictions were made either from broadly aligned features or from specific region-related features. Of course, these are individual examples drawn from a limited test set, and they should be treated as illustrative.
