# Supplementary material for: Within a Heartbeat: A Machine Learning Approach Using VCG for Heart Failure Assessment in Sinus Rhythm
Source: J Pers Med. 2026 Sep 17;16(9):480. doi: 10.3390/jpm16090480 (PMC13608557; doi:10.3390/jpm16090480)

normal vs. HFpEF

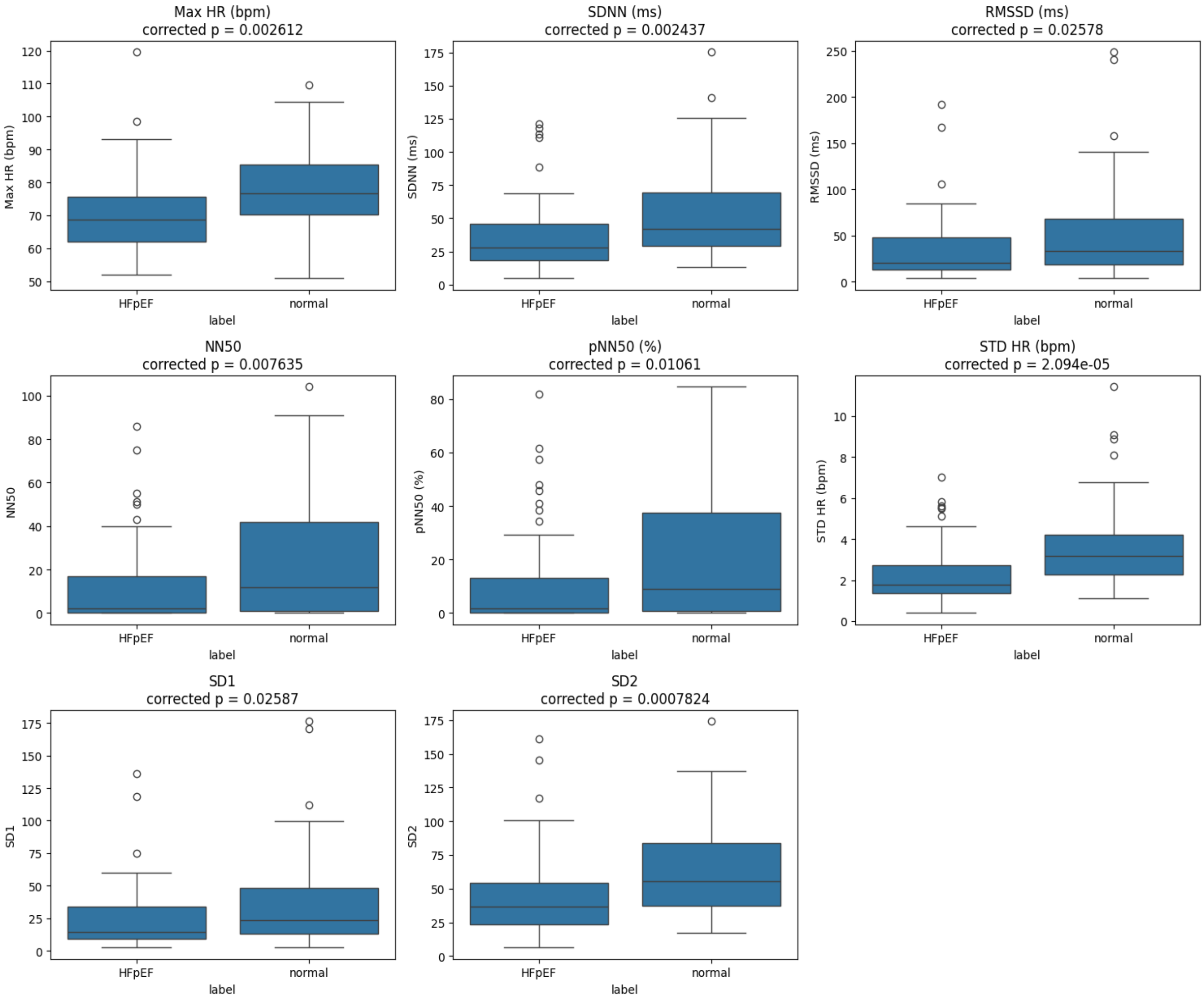

normal vs. HFrEF

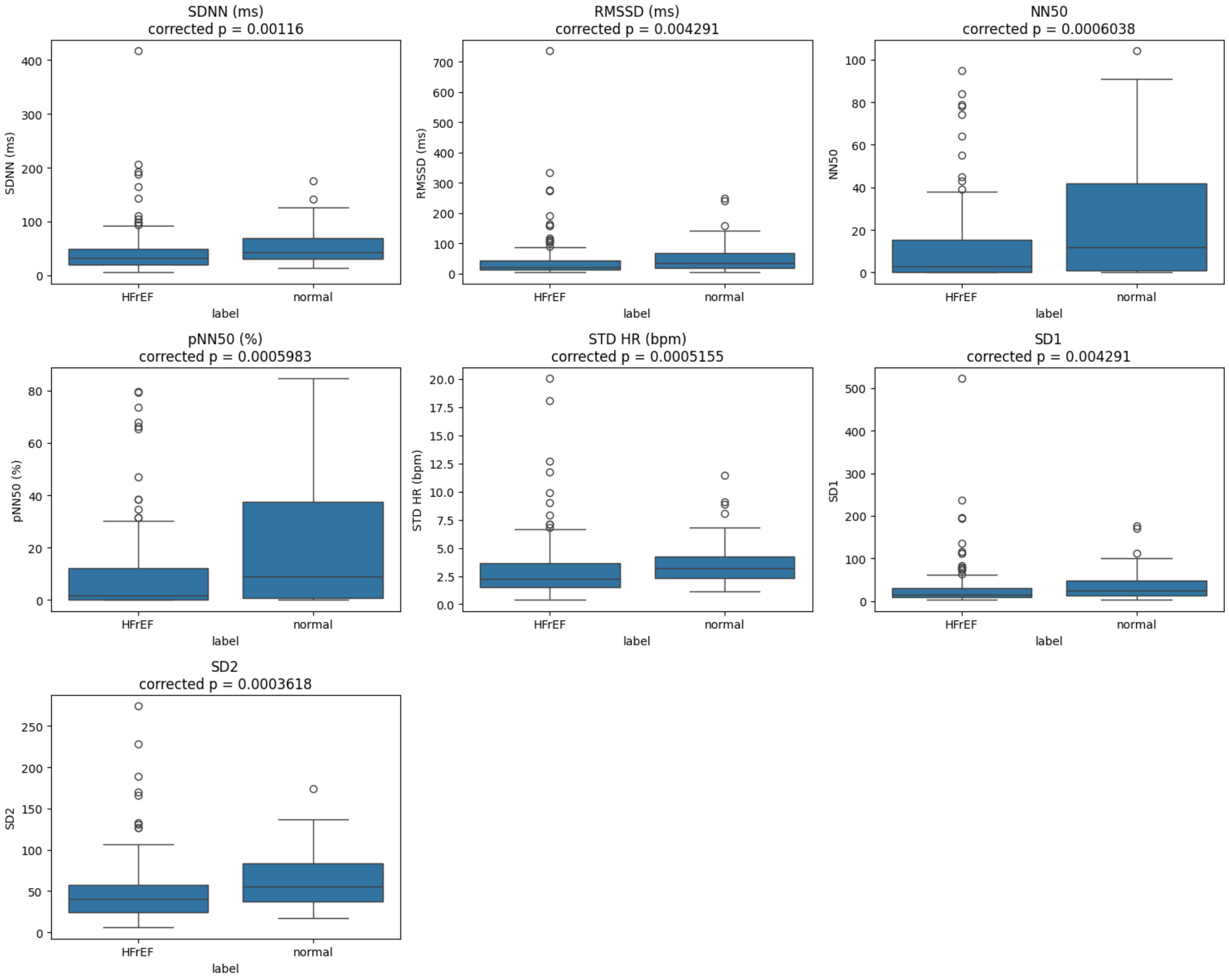

HFrEF vs. HFpEF

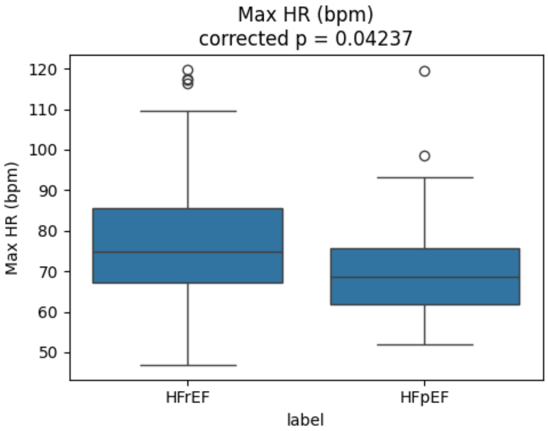

Supplement: Supplementary file 1 [file jpm-16-00480-s001.zip › Data S7.pdf]
